# Novel insights into the genomic basis of citrus canker based on the genome sequences of two strains of *Xanthomonas fuscans *subsp. *aurantifolii*

**DOI:** 10.1186/1471-2164-11-238

**Published:** 2010-04-13

**Authors:** Leandro M Moreira, Nalvo F Almeida, Neha Potnis, Luciano A Digiampietri, Said S Adi, Julio C Bortolossi, Ana C da Silva, Aline M da Silva, Fabrício E de Moraes, Julio C de Oliveira, Robson F de Souza, Agda P Facincani, André L Ferraz, Maria I Ferro, Luiz R Furlan, Daniele F Gimenez, Jeffrey B Jones, Elliot W Kitajima, Marcelo L Laia, Rui P Leite, Milton Y Nishiyama, Julio Rodrigues Neto, Letícia A Nociti, David J Norman, Eric H Ostroski, Haroldo A Pereira, Brian J Staskawicz, Renata I Tezza, Jesus A Ferro, Boris A Vinatzer, João C Setubal

**Affiliations:** 1 Departamento de Ciências Biológicas, Instituto de Ciências Exatas e Biológicas, Campus Morro do Cruzeiro, Universidade Federal de Ouro Preto, Ouro Preto, MG, Brazil; 2 Departamento de Bioquímica, Instituto de Química, Universidade de São Paulo, São Paulo, SP, Brazil; 3 Department of Plant Pathology, University of Florida, Gainesville, FL, USA; 4 Department of Plant Pathology, Physiology and Weed Sciences, Virginia Polytechnic Institute and State University, Blacksburg, VA, USA; 5 Departamento de Tecnologia, Faculdade de Ciências Agrárias e Veterinárias de Jaboticabal, UNESP - Univ. Estadual Paulista, Jaboticabal, SP, Brazil; 6 Departamento de Ciências Biológicas, Campus de Diadema, Universidade Federal de São Paulo, São Paulo, SP, Brazil; 7 Departamento de Melhoramento e Nutrição Animal, Faculdade de Medicina Veterinária e Zootecnia de Botucatu, UNESP - Univ. Estadual Paulista, SP, Brazil; 8 Departamento de Engenharia Florestal, Centro de Ciências Agroveterinárias, Universidade do Estado de Santa Catarina, Lages, SC, Brazil; 9 Allelyx Applied Genomics, Campinas, SP, Brazil; 10 Núcleo de apoio à pesquisa em microscopia eletrônica aplicada à pesquisa agropecuária, Escola Superior de Agricultura Luiz de Queiroz, Universidade de São Paulo, Piracicaba, SP, Brazil; 11 Laboratório de Bacteriologia Vegetal, Instituto Biológico Campinas, Campinas, SP, Brazil; 12 Escola de Artes, Ciências, e Humanidades, Universidade de São Paulo, São Paulo, SP, Brazil; 13 Faculdade de Computação, Universidade Federal do Mato Grosso do Sul, Campo Grande, MS, Brazil; 14 Laboratório de Bioinformática, Instituto de Computação, Universidade Estadual de Campinas, Campinas, SP, Brazil; 15 Virginia Bioinformatics Institute, Virginia Polytechnic Institute and State University, Blacksburg, VA, USA; 16 Department of Computer Science, Virginia Polytechnic Institute and State University, Blacksburg, VA, USA; 17 Instituto Agronômico do Paraná, Londrina, PR, Brazil; 18 Institute of Food and Agricultural Sciences, Mid-Florida Research & Education Center, University of Florida, Gainesville, FL, USA; 19 Department of Plant & Microbial Biology, University of California, Berkeley, Berkeley, CA, USA

## Abstract

**Background:**

Citrus canker is a disease that has severe economic impact on the citrus industry worldwide. There are three types of canker, called A, B, and C. The three types have different phenotypes and affect different citrus species. The causative agent for type A is *Xanthomonas citri *subsp. *citri*, whose genome sequence was made available in 2002. *Xanthomonas fuscans *subsp. *aurantifolii *strain B causes canker B and *Xanthomonas fuscans *subsp. *aurantifolii *strain C causes canker C.

**Results:**

We have sequenced the genomes of strains B and C to draft status. We have compared their genomic content to *X. citri *subsp. *citri *and to other *Xanthomonas *genomes, with special emphasis on type III secreted effector repertoires. In addition to *pthA*, already known to be present in all three citrus canker strains, two additional effector genes, *xopE3 *and *xopAI*, are also present in all three strains and are both located on the same putative genomic island. These two effector genes, along with one other effector-like gene in the same region, are thus good candidates for being pathogenicity factors on citrus. Numerous gene content differences also exist between the three cankers strains, which can be correlated with their different virulence and host range. Particular attention was placed on the analysis of genes involved in biofilm formation and quorum sensing, type IV secretion, flagellum synthesis and motility, lipopolysacharide synthesis, and on the gene *xacPNP*, which codes for a natriuretic protein.

**Conclusion:**

We have uncovered numerous commonalities and differences in gene content between the genomes of the pathogenic agents causing citrus canker A, B, and C and other *Xanthomonas *genomes. Molecular genetics can now be employed to determine the role of these genes in plant-microbe interactions. The gained knowledge will be instrumental for improving citrus canker control.

## Background

Citrus canker is a disease with worldwide distribution that has severe economic impact on the citrus industry [1,2]. Disease symptoms consist of water soaked lesions that develop into blisters, then pustules, and, finally, cankers. In severe cases, citrus canker can lead to defoliation and premature fruit drop [3]. Eradication of infected plants is the method of choice to control the disease where it is not yet endemic. When the disease is endemic, control is attempted by planting disease-free trees, limiting the spread between orchards, and using preventive copper sprays [4-6]. However, none of these measures controls citrus canker efficiently.

There are three types of citrus canker described in the literature: types A, B and C. Type A originated in Asia, probably in Southern China, Indonesia or India, and it is the type that is most widespread and causes the greatest economic damage [4,7]. The other two types have only been found in South America. Type B (or false canker) was originally identified in Argentina in 1923. This type is present only in Argentina, Paraguay, and Uruguay [8], whereas type C is limited to the state of São Paulo, Brazil [9].

The causal agent of canker A is *Xanthomonas citri *subsp. *citri *(which we abbreviate as XAC for reasons explained below). XAC causes disease on many citrus species, with *C. paradisi *(grapefruit) and *C. aurantifolia *(Mexican lime) being most susceptible in the field and *C. reticulata *(mandarin/tangerine) and *C. sinensis *(sweet orange) being relatively tolerant [10,11]. Importantly, no citrus species is resistant to XAC after artificial inoculation, suggesting that there is no true genetic resistance against XAC and that field tolerance is mainly due to variation in growth habit [3].

The genome of XAC strain 306 from Brazil was completely sequenced in 2002 [12] and compared to the genomes of *Xanthomonas *species that are pathogenic in other plants [13,14]. This comparative genomics approach has greatly accelerated the study of the molecular basis of pathogenicity and virulence of XAC. XAC has a *hrp/hrc *cluster coding for a type III secretion system (T3SS) that is used by the pathogen to inject virulence proteins, called effectors, into host cells. While several genes coding for putative effectors have been identified in the XAC genome, the single most important effector is PthA [3,15]. Even in the absence of the pathogen, PthA induces canker-like symptoms when transiently expressed in plants [16]. Deletion of *pthA *abolishes the ability of XAC to cause cankers [17]. Intriguingly, PthA induces cankers in all citrus species while it triggers plant immunity in other plant species, thus being the prime determinant of XAC specificity toward citrus [16,18,19].

Two variant forms of canker A have been described. The first was found in Southeast Asia in 1998 infecting *C. aurantifolia*. The pathogen was classified as XAC variant A* [19]. The second variant was isolated in 2003 in Southern Florida in *C. aurantifolia *and *C. macrophyla *(alemow), and was named *Xanthomonas citri *variant A^W ^[20]. A^W ^strains have been shown to be a sub-group within A* [20]. These strains are primarily pathogenic on *C. aurantifolia *and do not cause disease on *C. paradisi*, even after artificial inoculation [19,20]. A T3SS effector, called AvrGf1, was found to contribute to the exclusion of *C. paradisi *from the A* host range [21]. A recent study [22] suggests that A* strains (including A^W^) have a wider genetic diversity than the strains that cause A-type canker.

**Figure 1:**
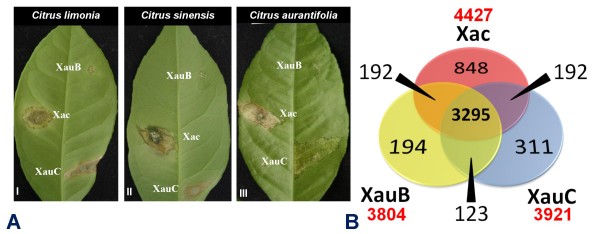
**Phenotype and Genotype**. **(A) **Disease symptoms caused by XAC, XauB, and XauC on leaves of three different citrus species. The lesions caused by XAC and XauB are similar but differ in size. XauC causes a hypersensitive response in *C. limonia *and *C. sinensis*, with *C. aurantifolia *as a true host. The pictures were taken 21 days after inoculation. **(B) **Venn diagram showing the numbers of protein-coding orthologous genes shared among the three strains.

Canker B is mostly restricted to *C. limon *(lemon), but has also been found in *C. sinensis *and in *C. paradisi *[8]. Its causal agent has been described as *X. fuscans *subsp. *aurantifolii *type B (which we abbreviate as XauB for reasons explained below). Even though symptoms are similar to canker A, they take longer to appear, perhaps reflecting the slower growth of XauB in culture when compared to XAC. Canker C has the same symptoms as type A, but, similarly to XAC A* and A^W^, it is restricted to *C. aurantifolia *and does not occur in *C. paradisi *[23]. The causal agent has been described as *X. fuscans *subsp. *aurantifolii *type C (which we abbreviate as XauC for reasons explained below). Fig. 1A summarizes observed phenotypes in three different citrus species. Recently, what appears to be a new type of *X. fuscans *subsp. *aurantifolii *infecting swingle citrumelo (*C. paradisi *Macf. × *Poncirus trifoliata *L. Raf.) has been reported in Brazil [24].

XAC, XauB and XauC have been compared phenotypically and analyzed phylogenetically. All three strains present polar flagella with perceptible motility when cultured in semi-solid media [25]. They all grow in the presence of lactose, manitol, and celobiose. However, only XAC is able to grow in the presence of maltose and aspartic acid, and it is also capable of pectate and gel hydrolysis [26]. XauB and XauC have little or no affinity for polyclonal antisera prepared against XAC, and XAC is susceptible to bacteriophages CP1 and CP2 while XauB and XauC are not [19]. It is notable that XauB has fastidious growth in culture media where both XAC and XauC grow well, for example in Agar nutrient and tryptophan-sucrose-agar media. All three grow well in media rich in glutamic acid [27]. Multilocus sequence typing and other molecular analyses [28-30] have shown that XauB and XauC are more closely related to each other than to XAC.

Under the rationale that the availability of the genome sequences and annotations of the causative agents of the B and C canker types can substantially improve our understanding of the genomic basis of the disease, we have sequenced the genomes of XauB and XauC to draft status. We have compared them with the genomes of XAC and other xanthomonads. Identified commonalities among the three canker genomes represent candidate genes that may help explain the differences between citrus canker and diseases caused by other xanthomonads. We have also identified numerous gene differences between the three citrus canker genomes. Some of these genes were previously shown to contribute to the virulence of XAC [31] and are thus primary candidates for explaining the higher virulence of XAC compared to XauB and XauC as well as the host range differences that exist between the three canker types.

### A note on species abbreviations

The organisms studied in this work do not have names that are universally accepted. At the time when its genome was sequenced, the accepted name for XAC was *Xanthomonas axonopodis *subsp. *citri*. In the meantime, *Xanthomonas citri *subsp. *citri *has been validly published as a name of this organism [32,33]. However, because the locus tag prefix of XAC genes is 'XAC' we opted for this abbreviation to avoid confusion along the text. Similarly, the causative agents for the B and C cankers are known by different names, but we use XauB and XauC as acronyms so that they are in agreement with their respective locus tag prefixes. For the B species we use the name *X. fuscans *subsp. *aurantifolii *type B and for the C species we use the name *X. fuscans *subsp. *aurantifolii *type C [33]. We refer to the three organisms collectively as the *citrus canker strains *(abbreviated by **CC strains/genomes**).

## Results and Discussion

Table 1 shows the genome features of the three canker strains. Even though we do not have complete genome sequences for XauB and XauC, alignments of their scaffolds to the XAC genome suggest that all three chromosomes are highly syntenic (Additional file 1: Fig. S1). Based on these alignments and on total length of contigs we estimate that we have obtained 94% of the XauB genome and 96% of the XauC genome. We estimate that the vast majority of remaining gaps in the two incomplete genomes is under 2 kbp. Fig. 1B shows that XAC shares 74% of its protein-coding genes with the other two strains. The fractions for XauB and XauC are 87% and 84%, respectively. The number of XAC-specific genes is much larger than the analogous number for XauB and XauC. Although this difference could be attributed to the incompleteness of the Xau genomes, we have hybridization results (see below) that suggest that many of these XAC-specific genes are indeed absent from the other two genomes under study.

**Table 1:** General features of XAC, XauB, and XauC genomes.

Feature	XAC	XauB*	XauC*
**genome**			

**size (bp)**	5,274,174	4,877,808	5,012,633

**# contigs**	3	239	351

**%GC**	64.7	64.9	64.8

**protein-coding genes**			

**total**	4,427	3,804	3,921

**with functional assignment**	2,779	2,694	2,728

**conserved hypothetical**	1,386	993	1,009

**hypothetical**	262	117	184

**RNAs**			

**rRNA operons**	2	2	2

**tRNAs**	54	51	51

For each strain the numbers are totals for the chromosome plus plasmids. The asterisk (*) denotes that the numbers for XauB and XauC come from their respective incompletely sequenced genomes.

XAC strain 306 has two plasmids, pXAC33 (34 kbp) and pXAC64 (65 kbp). Based on similarities between contig sequences and XAC plasmid sequences it appears that both XauB and XauC have plasmids. At least 46% of plasmid pXAC33 sequence is found in XauB contigs (46% of plasmid pXAC33 sequence is found in XauC contigs); at least 61% of plasmid pXAC64 sequence is found in XauB contigs (55% for XauC). We do not have enough sequence data to ascertain the exact number of plasmids in each Xau genome.

**Figure 2:**
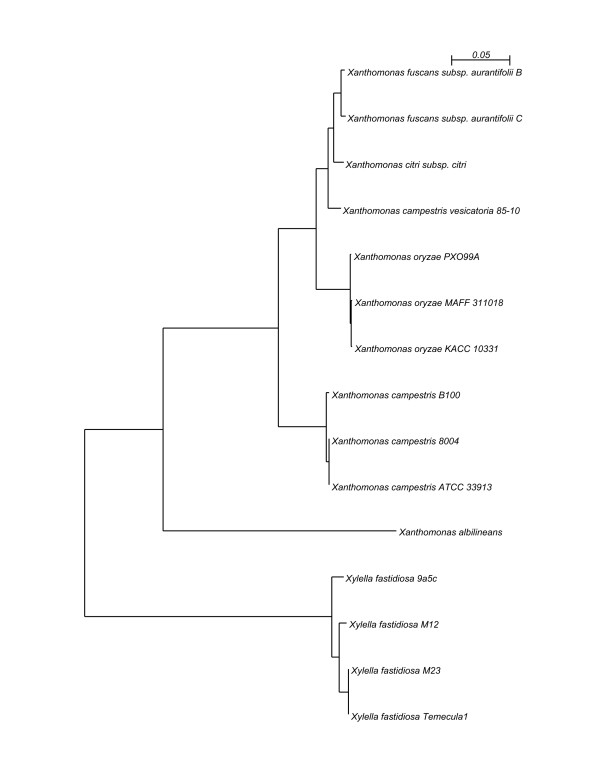
**Maximum likelihood tree of fully sequenced *Xanthomonas *and *Xylella *genomes**. The bootstrap support is 100% for all branches (100 bootstrap runs). Bar, number of amino acid substitutions per site.

The phylogenetic relationship of the three strains with respect to each other and to fully sequenced members of the *Xanthomonas *and *Xylella *genera based on their shared protein-coding genes is shown in Fig. 2. The tree shows that the three organisms under study form a well-defined group within the family *Xanthomonadaceae*. This tree is in agreement with trees of the *Xanthomonas *genus based on multilocus sequence analysis [34,35].

At http://bioinfo.facom.ufms.br/xanthomonas we provide an interactive tool that allows user-defined gene content comparisons among all sequenced *Xanthomonas *genomes.

### Genes shared by all three strains but not present in other *Xanthomonadaceae *species

XAC, XauB, and XauC have 65 families of orthologous genes specific to them when compared to all other fully sequenced members of the genera *Xanthomonas *and *Xylella *(Additional file 2: Table S2). Among these 65 families we have identified 11 syntenic blocks. Not surprisingly, almost half of these genes code for hypothetical proteins of unknown function. Of the genes with a predicted function the genes encoding the predicted effectors XopE3 (XAC3224) and XopAI (XAC3230) (discussed below) are especially noteworthy. The large number of genes coding for various kinds of transporters are also worth mentioning: four TonB dependent receptors including one with homology to the *Escherichia coli *receptor FepA, which is involved in transport of siderophores across the bacterial membrane [36] and seven ABC transporters, which might be used either for translocation of substrates from the citrus apoplast into the bacterial cell to provide nutrients for the pathogen, or, alternatively, for secretion of toxins (either bacterial toxins or expulsion of citrus metabolites toxic to the CC strains). An additional transporter specific to the three CC genomes (XAC3198) is an alkanesulfonate transporter substrate-binding subunit, which is reported to enable *E. coli *to use sulfonates other than taurine [37]. Besides transporters, several genes encode metabolic enzymes (an amidase, an urea amidolyase, a peptidase, and a nitrilotriacetate monooxygenase). This conspicuous presence of transporters and metabolic enzymes suggests that the CC strains might have adapted to specific metabolites present in the citrus apoplast. However, this will need to be confirmed experimentally by comparing growth of wild type strains and strains mutated in CC-specific genes in apoplastic fluid of citrus species and of other plant species. One other interesting gene present in all CC genomes is a gene coding for a methyl parathion hydrolase (XAC0726), predicted to degrade the insecticide methyl parathion [38,39]. Orthologs of this gene and other genes that degrade organophosphates are common in soil bacteria and in the soil-borne pathogen *Ralstonia solanacearum *but have not yet been found in any other foliar plant pathogen.

Some of these syntenic CC-specific regions are anomalous in terms of nucleotide composition as determined by the program AlienHunter [40] and may thus have been acquired by horizontal gene transfer.

### The three CC genomes have important differences in regard to their repertoires of type III secreted effectors

The *hrp/hrc *genes encoding the T3SS are basically the same and found in the same order in all three CC genomes. However, there are notable differences in the three putative T3SS-secreted effector repertoires.

A list of twenty-seven T3SS effector genes predicted in the genomes of the CC strains is shown in Table 2. Effectors are important determinants of virulence and host range in many plant pathogenic bacteria, in particular in *Xanthomonas sp. *and *Pseudomonas syringae *[41]. Comparison of effector repertoires between the three CC genomes and all other *Xanthomonas *genomes can thus give us important clues. The effector genes *avrBs2, xopL, xopQ*, and *xopX *are present in all three CC genomes, in all sequenced genomes of other *Xanthomonas *species, and in all *X. citri *and most *Xanthomonas *strains that were surveyed by PCR and hybridization for these genes by Hajri et al. [42]. These effectors thus belong to the *Xanthomonas *core set of effectors possibly important for pathogenicity on all plants. The putative effector genes *xopK, xopR*, and *xopZ *also belong to this group since they can be found in all sequenced *Xanthomonas *genomes. However, no data exist for these effectors in regard to other *Xanthomonas *strains [42]. The effector genes *xopI, xopV, xopAD*, and *xopAK *are present in all three CC genomes and in several, but not all, sequenced *Xanthomonas *genomes. These effectors, therefore, might contribute to disease in some plant species while they might trigger immunity in others.

**Table 2:** Putative effectors found in the XAC, XauB, and XauC genome sequences.

Effector family	XAC	XauB	XauC	Pfam: functional/structural domain	Effectors
**Candidate effectors common to XAC, XauB, and XAUC**					

**AvrBs2**	XAC0076	XAUB_16770	XAUC_23650	Glycerophosphoryl diester phosphodiesterase	AvrBs2 from *X. campestris pv. vesicatoria *[119]

**AvrBs3**	XACa0022 (pthA1) XACa0039 (pthA2) XACb0015 (pthA3) XACb0065 (pthA4)	XAUB_40130 XAUB_28490	XAUC_22430 XAUC_24060 XAUC_09900 XAUC_43080	Transcriptional activator, nuclear localization	PthA [17]

**XopE1 (avrXacE1, hopX, avrPphE)**	XAC0286	XAUB_37010	XAUC_37580	Putative transglutaminase	AvrXacE1, XopE1 from *X. campestris pv. vesicatoria *[120]

**XopE3 (avrXacE2, hopX, avrPphE)**	XAC3224	XAUB_14680	XAUC_00040	Putative transglutaminase	AvrXacE2 [121]

**XopF2**	XAC2785 Ψ	XAUB_07540 Ψ	XAUC_21000 Ψ		XopF2 [122]

**XopI**	XAC0754	XAUB_39080	XAUC_07100	F-box protein	*X. campestris pv. vesicatoria *[120]

**XopK**	XAC3085	XAUB_34090	XAUC_12520		Identified in Xoo by cya assay [123]

**XopL**	XAC3090	XAUB_34130	XAUC_02900/12488 Ψ	LRR protein	[121]

**XopQ (hopQ1)**	XAC4333	XAUB_10220	XAUC_14670	Inosine uridine nucleoside N-ribohydrolase	[124]

**XopR**	XAC0277	XAUB_36920	XAUC_37490		Identified in Xoo by cya assay [123]

**XopV**	XAC0601	XAUB_23140	XAUC_21260		Identified in Xoo by cya assay [123]

**XopX (HolPsyAE)**	XAC0543	XAUB_14760	XAUC_20690		[125]

**XopZ (HopAS, AWR)**	XAC2009	XAUB_11532/13710 Ψ	XAUC_25915		[123]

**XopAD (skwp, RSc3401)**	XAC4213	XAUB_02510	XAUC_34870	SKWP repeat protein	Skwp from *Ralstonia *[126]

**XopAI (HopO1 (HopPtoO, HopPtoS), HopAI1 (HolPtoAI))**	XAC3230	XAUB_26830	XAUC_23780	ADP-ribosyltransferase	[120]

**XopAK (HopAK1 (HopPtoK, HolPtoAB)C terminal domain)**	XAC3666	XAUB_02580	XAUC_32490		Not confirmed to be effector in *Xanthomonas*; homolog of effector in *Pseudomonas*

**HrpW (PopW)**	XAC2922	XAUB_19460 (associated with hrp cluster)	XAUC_20020 (associated with hrp cluster)	Pectate lyase, may not be T3SE	[127]

**Candidate effectors present in XAC and XauB BUT ABSENT in XauC**					

**XopE2 (avrXacE3, avrXccE1)**	XACb0011	XAUB_31660	-	Putative transglutaminase	XopE2 found in another C strain [120]

**XopN (hopAU1)**	XAC2786	XAUB_07520	-	ARM/HEAT repeat	[51]

**XopP**	XAC1208	XAUB_06720	-		[124]

**XopAE (HpaF/G/PopC)**	XAC0393	XAUB_19500	-	LRR protein	Xcv8510 [52]

**Candidate effectors present in XauB and XauC BUT ABSENT from XAC**					

**XopB (hopD1, avrPphD1)**	-	XAUB_09070/14842 Ψ	XAUC_00260		[128]

**XopE4 (HopX)**	-	XAUB_23330	XAUC_31730		New class introduced

**XopJ (AvrXccB)**	-	XAUB_20830	XAUC_08850	C55-family cysteine protease or Ser/Thr acetyltransferase	[129]

**XopAF (avrXv3, HopAF1 (HopPtoJ))**	-	XAUB_02310	XAUC_00300		[130]

**XopAG (AvrGf1, HopG1 (HopPtoG). HolPtoW)**	-	XAUB_03570 Ψ	XAUC_04910		AvrGf1 [21]

**Candidate effectors present only in XauC**					

**XopF1 (Hpa4)**	-	-	XAUC_20060 Ψ		[124]

Some of the effectors appear to be pseudogenes (indicated by a Ψ). In column 'effector family' we provide the standard effector name along with the names of related proteins belonging to the same family; in column 'effectors' we provide the reference where the effector indicated was characterized.

As already mentioned, PthA is well known to be an important *X. citri *effector that plays an essential role in citrus canker, while limiting the host range of CC strains to citrus because it triggers immunity in all other tested plant species (see references above). The *pthA *gene is a member of the *avrBS3 *family of effector genes, members of which are present in most *Xanthomonas *genomes and in some *R. solanacearum *genomes [43]. However, only PthA is known to induce citrus canker. Besides *pthA *(XACb0065), three paralogs of *pthA *are also present in the XAC genome (XACa0022, XACa0039, and XACb0015). All four copies are found on plasmids. The three paralogs do not seem to play an important role in citrus canker [18]. We found two *pthA *homologs in the XauB genome (XAUB_40130 and XAUB_28490) and two in the XauC genome (XAUC_22430 and XAUC_24060/XAUC_09900 [the latter is a single gene with halves in different contigs]). Not all of these genes have been completely assembled due to the repetitive regions found in *avrBS3 *family members. However, El Yacoubi et al. [44] previously assembled a *pthA *homolog (*pthB *[GenBank: 2657482]) from the pXcB plasmid [GenBank: NC_005240] of a XauB strain with the same repeat copy number (i.e. 17.5) as *pthA*, and Al-Saadi et al. [17] sequenced and assembled another homolog (*pthC *[GenBank: EF473088]) from a XauC strain. These genes functionally complemented a *pthA *deletion in XAC without affecting host range [17]. The XAUC_22430 gene has 99% nucleotide identity to *pthC *and thus probably corresponds to *pthC *and would be the functional *pthA *homolog of XauC. We do not have enough data to confidently report on the repeat copy number of the other three Xau *pth *homologs, but a phylogenetic analysis (see below) suggests that XAUB_28490 is the functional *pthA *homolog of XauB.

**Figure 3:**
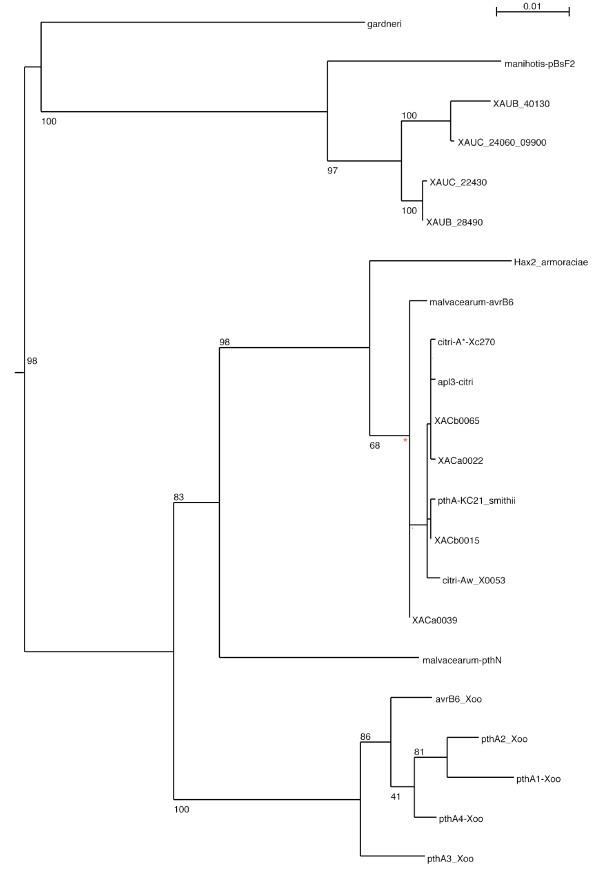
**Maximum likelihood tree of *Xanthomonas pth *genes**. The sequences used to build the tree had their tandem repeat portions masked before alignment. The root position was obtained from a preliminary tree that included as an outgroup a *Ralstonia solanacearum pth *gene [GenBank: CAD15517.1]. Full information about the *pth *gene sequences used is given in additional file 6 (Table S6). The numbers on branches represent bootstrap support (from 100 bootstrap runs). The bootstrap values obtained for the clade indicated by the asterisk * are given in additional file 7 (Fig. S7). Bar, number of amino acid substitutions per site.

To get insight into the evolution of the *pthA/avrBS3 *family, a phylogenetic analysis of all available *avrBS3 *family members in the genus *Xanthomonas *was performed (Fig. 3). The tree shows that the four XAC *pth *genes group together, while the XauB and XauC genes form two families, with one member from each strain participating in each family. Moreover, the XauB and XauC *pth *genes group separately from the XAC genes, with good bootstrap support, which is a surprising result when compared with the species tree (Fig. 2). The genes that flank the *pth *copies in XauB do not have XAC genes as their best BLAST [45] hits. In particular, the two genes upstream of XAUB_40130, XAUB_40110 and XAUB_40120, do not match any *Xanthomonas *genes; instead, their best BLAST hits (e-value 10^-102 ^and 10^-99^, respectively) are gene sequences from *Burkholderia pseudomallei *NCTC 13177. (In XauC the *pth *regions are too fragmented to allow genomic context determination.) We derive the following conclusions from these results: An ancestor of the current XauB and XauC strains already had two different copies of the *pth *genes; hence the existence of the two noted families. The fact that in XAC the four copies are nearly identical, the fact that the Xau and XAC *pth *genes group in a way distinctively different from the species grouping, and the fact that the genomic context in which the XAC *pth *genes are found is different from that of XauB leads us to believe that XAC acquired its *pth *genes by a different route and that their duplication is more recent when compared to the XauB and XauC *pth *duplications. The ability to cause citrus canker may thus have independently evolved by XAC and by XauB and XauC, and may represent an example of convergent evolution. Al-Saadi et al. [17], based on a neighbor-joining phylogeny of *pth *genes that included the *pthB *and *pthC *sequences, have come to the same conclusion.

### Effectors XopAI and XopE3 may play a role in citrus canker

A comparison of effectors present in all three CC strains with those present in fully sequenced *Xanthomonas *species, and data from the study by Hajri et al. [42], suggest that two additional putative effectors may play a special role in citrus canker. These are XopAI and XopE3. Both are present in all three CC genomes.

The putative effector xopAI is not found in any other sequenced *Xanthomonas *species and it was not included in the Hajri et al. [42] analysis. We do have evidence that it is present in *Xanthomonas vesicatoria *str. 1111 (Potnis et al., unpublished). Interestingly, the C-terminal region of XopAI has similarity to predicted ADP-ribosyl transferase domains of the effector HopO1-1 of *Pseudomonas syringae *and of hypothetical proteins in *Acidovorax citrulli*, *Ralstonia solanacearum*, and other bacteria. The N-terminus has high similarity to the N-terminus of the effector XopE2 of *X. campestris *pv. *vesicatoria *85-10 as well the N-termini of a number of other *Xanthomonas *and *Pseudomonas syringae *effectors (more on the N-terminal region of *xopAI *below).

XopE3 belongs to the HopX/AvrPphE family of effectors. Effectors belonging to this family have been found in diverse phytopathogenic bacteria including *Ralstonia, Pseudomonas, Acidovorax*, and *Xanthomonas*, suggesting their conserved role in virulence on a wide range of hosts. Sequences from this family have similarity to the transglutaminase superfamily of enzymes, which are responsible for modification of host proteins [46]. The HopX/AvrPphE effector from *Pseudomonas syringae *has been shown to be involved in host protein proteolysis, thereby suppressing host defenses [46,47]. In xanthomonads, multiple effectors belonging to this group have been found, such as *xopE1, xopE2, xopE3, xopE4*. *XopE1 *and *xopE2 *have been found in most of the xanthomonads. *XopE3 *effector gene homologs have been found by PCR and dot-blot hybridization methods in some *Xanthomonas axonopodis *strains belonging to the *alfalfae*, *anacardii*, *glycines*, *phaseoli*, *malvacearum*, *fuscans*, *mangiferae, indicae*, and *citrumelo *pathovars [42]. However, sequences of *xopE3 *from these strains could not be compared against homologs from CC strains since sequence data from the *X. axonopodis *strains mentioned are not currently available. Phylogenetic analysis of *hopX *orthologs shows that the *xopE3 *effector genes found in the CC strains group together with *hopX1 *effector genes from pseudomonads (data not shown).

Although all *hopX *orthologs show conservation of the catalytic triad (Cys, His, Asp residues) as well as the conserved domain "GRGN" N-terminal to the triad, the region C-terminal to the triad shows high degree of variability. This variable region has been hypothesized to be responsible for targeting different host proteins [46]. In fact, while some AvrPphE (hopX) alleles from *P. syringae *pv. *phaseolicola *strains trigger gene for gene disease resistance in some bean cultivars, other alleles were shown to be virulent on these same cultivars. Amino acid differences in the C-terminal region of AvrPphE were identified between alleles [48]. Similarly, comparing *XopE3 *homologs from different strains at the amino acid level and their corresponding reactions on different hosts might give clues regarding the variable C-terminal domains of *XopE3 *family members and might determine whether this variability is responsible for targeting different proteins in different host species.

**Figure 4:**
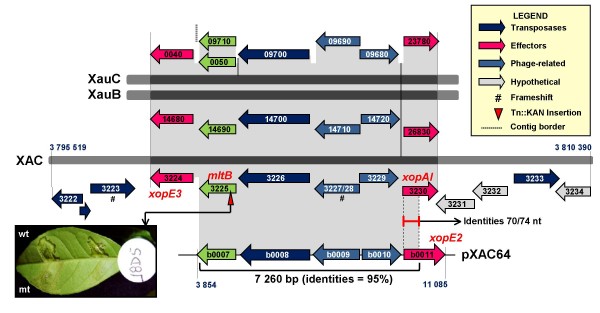
**Region containing citrus-canker specific effector genes *xopE3 *and *xopAI***. The XAC region depicted is hypothesized to be a genomic island [14]. The central parts of this region (gray areas) are CC-specific, being present in both XauB and XauC as well as in XAC plasmid pXAC64. Gene *mltB *is not an effector but plays a role in Type III secretion.

Both *xopE3 *and *xopAI *belong to an interesting XAC chromosomal region of approximately 15 kbp in size (Fig. 4) that has been hypothesized to be a genomic island [14]. An alignment of the XAC chromosome sequence with the chromosome sequences of *X. campestris *pv. *campestris *str. ATCC33913 and *X. oryzae *pv. *oryzae *str. PX099A strongly suggests that this region is an insertion (data not shown). The presence of three transposase genes and two phage-related genes in the region provides additional evidence for this hypothesis. The central part of this region (7 kbp) duplicates a region found in XAC plasmid pXAC64 (Fig. 4), suggesting a chromosome-plasmid DNA exchange. In the plasmid we find the effector gene *xopE2 *(XACb0011), which - as described above - shares its N-terminal region with *xopAI *(XAC3230) (Fig. 4). Transposons and phage elements in this region might thus have been responsible for a shuffling process, described as terminal reassortment [49], resulting in the novel effector gene *xopAI*. Although we can characterize this region completely only in XAC, XauB and XauC contigs contain the most important elements of this region (Fig. 4). Next to *xopE3 *(XAC3224) we find gene XAC3225, whose product is annotated as tranglycosylase *mltB*. This gene has strong similarity (e-value 10^-133^, 100% coverage) to *hopAJ1 *from*P. syringae *pv. *tomato *strain DC3000, where it is annotated as a T3SS helper protein. Although the *hopAJ1 *gene is not itself a T3SS substrate, it contributes to effector translocation [50]. A mutant with a deletion of XAC3225 has reduced ability to cause canker (mutant phenotypes include a reduction in water soaking, hyperplasia, and necrosis compared to wild type) [31]. We thus conclude that the effector and effector-related genes in this region probably play an important role in citrus canker.

### Additional differences in effector repertoires among CC genomes

In addition to the *pth *differences noted above, other effectors that distinguish the XAC genome from the two Xau genomes are *XopB, XopE4, XopJ (AvrXccB), XopAF (avrXv3)*, and *XopAG*, which are all present in both Xau genomes but absent from XAC strain 306. (AvrXccB homologs were found in two XAC strains by Hajri et al. [42].) The absence of these effectors from XAC strain 306 raises the possibility that these effectors might be responsible for limiting the host range of both B and C strains. Interestingly, XauB and XauC strains both contain *xopAG*, an effector gene belonging to the same effector family as *avrGf1 *from *X. citri A*^*w*^, which has been shown to be responsible for triggering a hypersensitive defense response in *C. paradisi *(grapefruit) [21]. The *xopAG *gene from the B and C genomes shows 44% identity to *avrGf1 *at the amino acid level. The XauB and and XauC genes are almost identical to each other, with one important difference: in XauB *xopAG *is interrupted by a transposon. Therefore, the incompatibility between XauC and grapefruit and the ability of XauB to cause disease in grapefruit could be explained by this single gene difference. The *xopE4 *DNA sequence is identical in the two Xau genomes and has similarity to *avrXacE3 *but only with 31% identity at the amino acid level; this is why we named this gene *xopE4 *instead of *xopE2*. Unlike other XopE family members, XopE4 does not have a predicted myristoylation site, suggesting that it may not be targeted to the cell membrane as the other XopE family members.

Presence of an additional effector gene, the avirulence gene *avrXccA2*, has been shown in some *X. aurantifolii *B (CFBP3528, CFBP3530) and *X. aurantifolii *C (CFBP2866) strains by hybridization and PCR analysis [42]. However, this avirulence gene was not found in the two sequenced Xau genomes. A homolog of the effector *xopF1 *(XAUC_20070) was found only in the *XauC *strain. It is located in a 5-kbp region that lies between the T3SS genes *hrpW *(XAUC_20020) and *hpa3 *(XAUC_20080). The same two genes are adjacent in XauB. Two transposases are present in this region, and the sequence of *xopF1 *has a frameshift, suggesting that this gene is likely the result of a recent insertion and is not active.

There are four effector genes present in the XAC and XauB genomes that have not been found in the genome of XauC: *xopE2, xopN, xopP*, and *xopAE*. These effectors could explain the wider host range of XAC and XauB compared to XauC, assuming a virulence activity of these effectors on citrus species. XopN has been shown to interact with the plant protein TARK1 and to interfere with immunity triggered by pathogen-associated molecular patterns (PAMP-triggered immunity) [51]. Further experiments are required to determine the possible role of XopN in extending host range to lemon, grapefruit and sweet orange. Another effector that could have a similar role is XopAE (a hpaF/PopC homolog) [52,53].

The harpin-like protein HrpW with a pectate lyase domain is present in all CC strains. In the sequenced XAC genome, it is not associated with the T3SS gene cluster, whereas in the genomes of XauB and XauC it is. The role of harpin-like proteins like HrpW as virulence factors or T3SS accessory proteins has not yet been determined in the *Xanthomonas *genus. Experiments will need to be performed to confirm translocation of the above putative effectors and their putative function as virulence or avirulence genes.

### XAC-specific genes and genomic regions with respect to XauB and XauC

**Figure 5:**
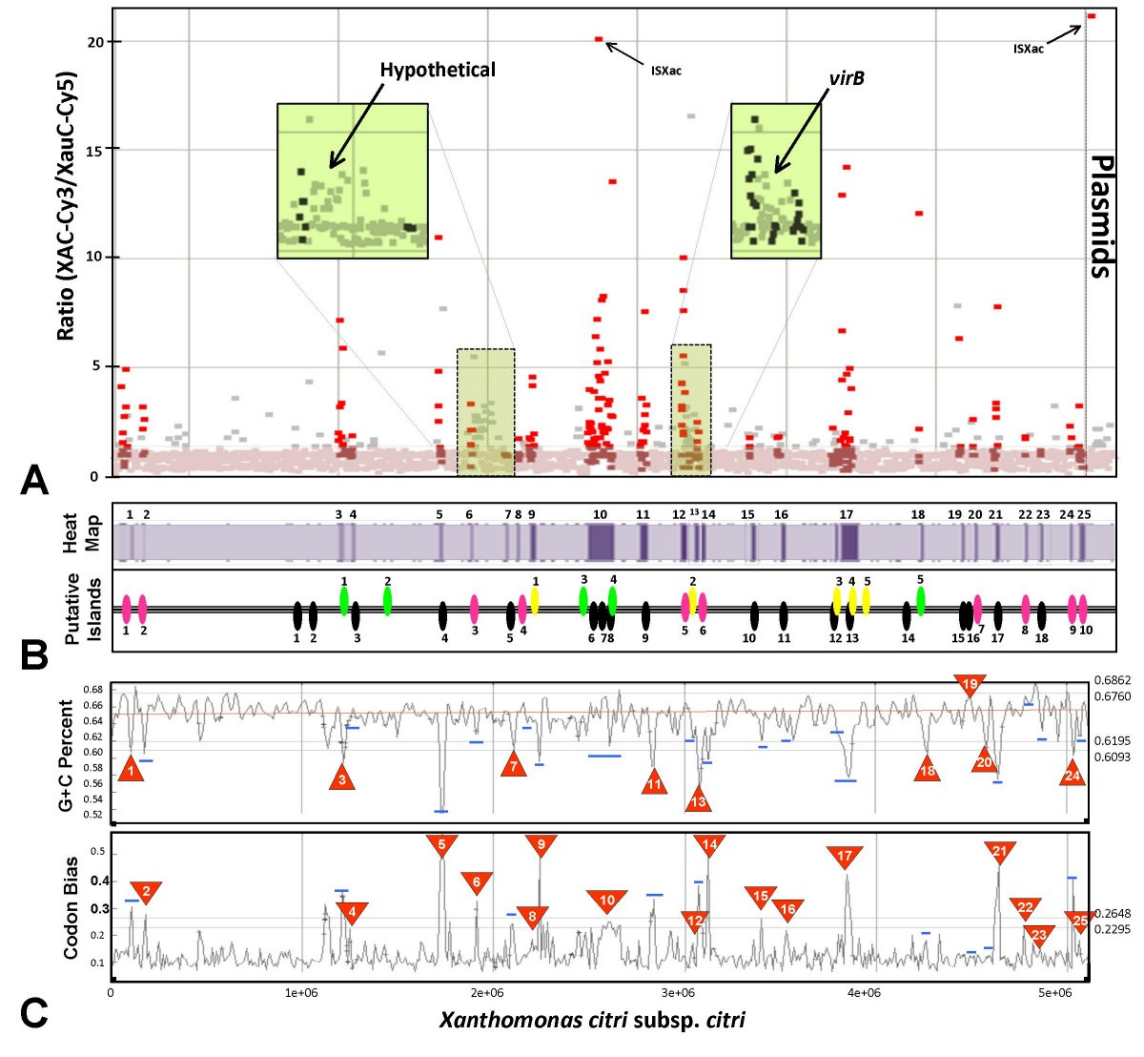
**Genomotyping and similarity analysis between the genomes of XAC and XauC based on DNA hybridization and matching of contig sequences**. **(A) **The hybridization results showed that 2,486 CDSs (out of a total 2,760) gave a hybridization signal greater than the estimated background noise. Of the 2,486 CDSs, 2,341 (94.2%) seem to be present in XauC (ratio Cy3/Cy5 between 0 and 1.5). The remaining 145 CDSs (5.8%) seem specific to XAC. **(B) **Of these 145, most (101 = 70%) belong to regions previously described as putative genomic islands in XAC (black ovals: [14]; green ovals: [106]; yellow ovals: [31]; the oval numbering corresponds to the original publication numbering). Pink ovals mark other nonsimilar regions between the genomes of XAC and XauC. **(C) **The two bottommost graphs (G+C and codon bias) show variation of these two metrics along the XAC genome, thus presenting evidence for the putative genomic islands denoted by ovals. The numbered triangles correspond to the XAC-specific regions based on DNA hybridization. Blue horizontal bars simply denote regions in these graphs that correspond to ovals not associated with XAC-specific regions. The Heat Map shows gene groups that yielded differential hybridization signals, the vast majority of which correspond to regions marked by ovals. The zoomed-in regions in the upper diagram show genes with differential hybridization signals but that are in regions shared by the two genomes. The XAC and XauC genes in these regions have less similarity (from 42 to 68% identity) between them than other shared genes.

We compared the XAC genome to the XauB and XauC genomes both computationally and by doing experimental whole genome hybridizations. Results are summarized in Fig. 5. The results of the two methods were consistent. In the following sections we focus on regions and genes specific to XAC with respect to XauB and XauC according to these results.

We have identified 25 groups of at least four consecutive genes that we term XAC-specific regions (XACSR) [Fig. 5, additional file 3 (Table S3), and additional file 4 (Fig. S4)]. Nearly all regions contain or are flanked by transposition elements or phage-related genes, suggesting that they could be the result of lateral transfer.

### Several genomic differences are related to biofilm formation and quorum sensing

Xanthan gum is an exopolysaccharide that plays an important role in biofilm formation and hence in virulence of pathogens of the *Xanthomonadaceae *family [54-56]. Moreover, the synthesis of xanthan gum is regulated by variation in sugar concentration in the culture medium and by the activation of regulatory *rpf *genes [57,58]. These genes are also responsible for the synthesis of diffusible signal factors, fundamental molecules for quorum sensing processes [59,60]. Both Xau genomes contain an identical xanthan gum operon (XauC: XAUC_26940-27060; XauB: XAUB_007400-007410 and XAUB_10560-10450). The Xau genomes contain gene *rpfH *(XAUB_10500 and XAUC_27010), but this gene is not found in XAC. Gene *rpfI*, present in the xanthan gum operon of *X. campestris *pv. *campestris *strain ATCC33913, is absent from all three CC genomes.

**Figure 6:**
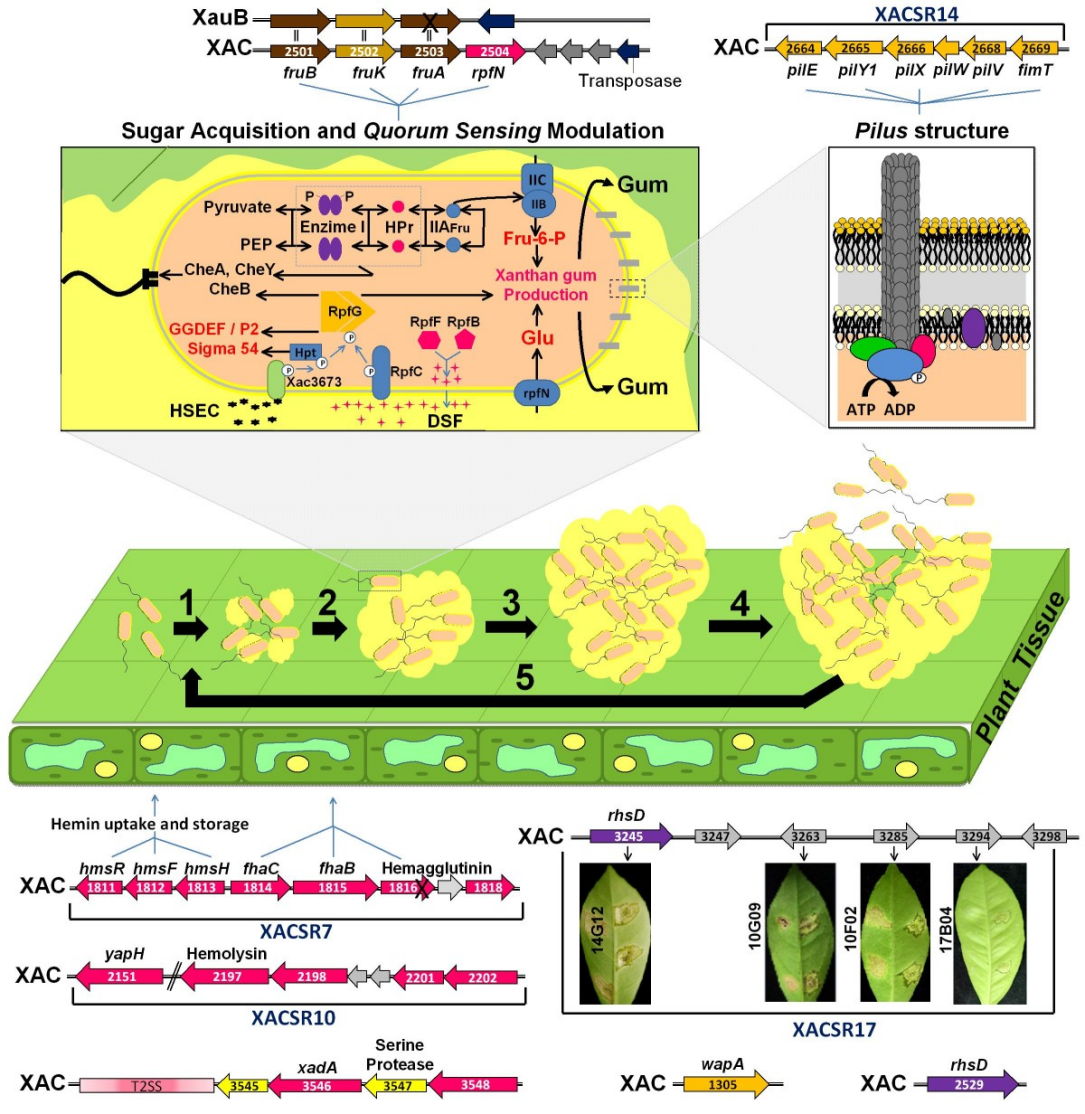
**Models for sugar acquisition, quorum sensing modulation, and biofilm formation and the XAC-specific genes and regions related to these processes**. Region XACSR17 (lower right corner) contains four genes that when mutated (separately) cause decrease in biofilm activity and virulence, as indicated in the leaf photos. The photo labels are mutant strain identifiers.

XAC and the two Xau genomes contain the xanthomonadin biosynthesis regulon as well as sugar metabolism genes. XauB however presents some important variations that may explain its *in planta *and in culture fastidious phenotype, when compared to both XAC and XauC. Differences were found in the phosphotransferase system (PTS-Fru), which specializes in internalization of fructose, and in the *rpfN *gene, a sugar porin, which is regulated by *rpf *genes, which also regulate xanthan gum synthesis [57]. The importance of the PTS-Fru system and of the sugar porin encoded by the *rpfN *gene for growth and pathogenicity of certain bacteria has been reported in the literature: PTS-Fru mutants of *Spiroplasma citri*, causative agent of citrus stubborn disease, are reduced in virulence [61-64], and *rpfN *mutants of *Xanthomonas campestris *pv. *campestris *show an increase in the level of polygalacturonate lyase [65], an important pathogenicity factor in bacterial plant pathogens. This result shows that the absence of the sugar porin could cause lack of carbohydrate uptake, therefore inducing the synthesis of cell wall degrading enzymes, in order to increase sugar supply [58,66]. In XAC the PTS system, encoded by genes *fruBKA *and *rpfN*, is organized in one single genomic region (XAC2501-2504) (Fig. 6). XauC presents the same organization (XAUC_01750-01780). In XauB *fruBKA *corresponds to XAUB_05120, XAUB_05110 and XAUB_05100 respectively, but *rpfN *was not found. In addition, the *fruA *gene sequence contains a frameshift, indicating that it may have become a pseudogene. XauB needs a culture medium with glutamic acid [27], possibly because it is used by the bacterium as an alternative carbon source. *Xylella fastidiosa *9a5c, which also lacks the PTS system and the *rpfN *gene, is also fastidious [67].

Experiments were carried out to compare cellular growth and xanthan gum production in all three organisms under study. Results (Table 3) confirm the fastidiousness of XauB, whereas XAC and XauC have similar cellular mass values. We also determined that XAC produces more than twice as much gum as XauC and almost three times as much as XauB. We believe this variation is due to the XauB deficiencies in the *rpfN *and PTS-Fru genes discussed above. On the other hand, this result also suggests that the lack of the *rpfH *and *rpfI *genes in XAC, caused by the presence of transposition elements [68], does not influence the gum production capability of XAC. The fastidiousness of XauB would thus be related to the low gum production and the consequent decrease in biofilm formation together with the fact that it needs an additional substrate source (glutamate) to support its growth in culture.

**Table 3:** Xanthan gum production.

Strain	Pellet weight mg/ml (sd)	Gum production mg/ml (sd)
XAC	2,57 (± 0,25)	3,93 (± 0,83)
XauB	1,70 (± 0,20)	1,62 (± 0,50)
XauC	2,35 (± 0,92)	1,82 (± 0,33)

sd - Standard deviation

In addition to the differences noted above, five additional XAC-specific regions (XACSR7, XACSR9, XACSR10, XACSR14 and XACSR17) may be related to its greater biofilm-formation capability when compared to XauB and XauC. Several of the genes in these regions facilitate adhesion in a process mediated by hemagglutinin [69].

XACSR7 contains two hemin storage system genes, *hmsF *and *hmsH*, and hemagglutinin coding genes (XAC1811-1816). Genes involved with acquisition and storage of hemin groups (*hmsRFH*) and type I secretion system genes (*fhaC*), and their secreted hemagglutinin (*fhaB*), are found *in tandem *and flanked by a tRNA^*R *^in the XAC genome (Fig. 6). In *Yersinia pestis *the *hms *genes are present in a cluster (the *pgm *cluster) related to temperature-dependent storage of hemin as well as expression of a number of other physiological characteristics [70]. Mutations in these genes cause drastic decrease in *Yersinia *growth, preventing it from colonizing its point of entrance in infected flies (bucal orifice) [71,72]. These genes also play a role in exopolysacharide synthesis, and reduction in biolfilm formation has also been observed in these mutants in *Yersinia *[73]. Among all sequenced *xanthomonads *only XAC and *X. oryzae *have these genes. In both cases they are similar to (35 to 53% identity at the amino acid level) and syntenic with their homologs in *Yersinia, E. coli *K-12 and *Erwinia carotovora *(data not shown).

Recent work [74] describing mutations in the genes that code for hemagglutinin in *Xylella fastidiosa *strain Temecula (Pierce's disease) has shown that biofilm composition and virulence (adhesion and colonization) were affected in the mutants. This is consistent with results in XAC [69]. This is evidence that the apparent absence of these genes in XauB and XauC might have the same effect (Fig. 6).

XACSR9 contains 19 genes. One of them (XAC1918) is a hemolysin-related gene. In enterobacteria hemolysins are an important virulence factor that are associated with proteins related to biofilm formation [75,76]. The protein encoded by this particular gene interacts with VirD4, a Type IV secretion system component [77], which in turn may play a role in biofilm formation and cell aggregation, as observed for *E. coli *[78].

XACSR10 contains several noteworthy genes. Gene XAC2151 codes for the YapH protein. Its homolog (XOO2380, 84% identity at the amino acid level) in *X. oryzae *pv. *oryzae *KACC10331 when mutated drastically reduced the pathogen adhesion to plant tissue, thus decreasing its virulence [79]; in addition a homolog of this gene in *X. fuscans *subsp. *fuscans *CFBP4834-R (the causative agent of bacterial blight of bean, *Phaseolus vulgaris*), was required for adhesion to seed, leaves, and abiotic surfaces [80]. XAC2197 and XAC2198 code for hemolysin-type calcium binding proteins, whereas XAC2201 and XAC2202 code for hemolysin secretion protein D (HlyD) and hemolysin secretion protein B (HlyB), respectively. The latter four genes do not have *Xanthomonas *matches in the sequence databases; they are similar instead to protein sequences from *Acidovorax*, *Xylella *and *Pseudomonas *species. The best hits are from *Acidovorax avenae *subsp. *avenae *ATCC19860, which is also a plant pathogen.

XACSR14 contains genes related to the type IV pilus-dependent system (Fig. 6). This system takes part in several processes, including adhesion, motility, microcolony formation, and protease secretion [81]. In *Xylella fastidiosa *functional studies of these genes have shown that they are crucial for the host colonization process [82-86].

XACSR17 is also related to biofilm formation. Laia et al. [31] observed decrease in biofilm activity and virulence in four mutants with changes in this region (XAC3245-14G01/14G12, XAC3263-10G07/10G09, XAC3285-10F02 and XAC3294-17B04) (Fig. 6). The only mutated gene with functional assignment is *rhsD *(XAC3245). This gene has been described as coding for a membrane protein related to adhesion [69]. In *Xanthomonas campestris *pv. *campestris *a RhsD protein has been found in the outer membrane vesicle associated with other virulence-associated proteins, such as HrpA/F/X/B4, HrcU, AvrBs1 and AvrBs2 [87]. XAC contains a paralog of this gene (XAC2529), but it was not found in the Xau genomes either.

Gene *wapA *(XAC1305), not part of any XAC-specific region, and which is an adhesion facilitator by way of hemagglutinins [69], was not found in either of the Xau genomes.

### XauB contains T4SS gene clusters similar to those found in *Ralstonia solanacearum *and in *Agrobacterium tumefaciens*

**Figure 7:**
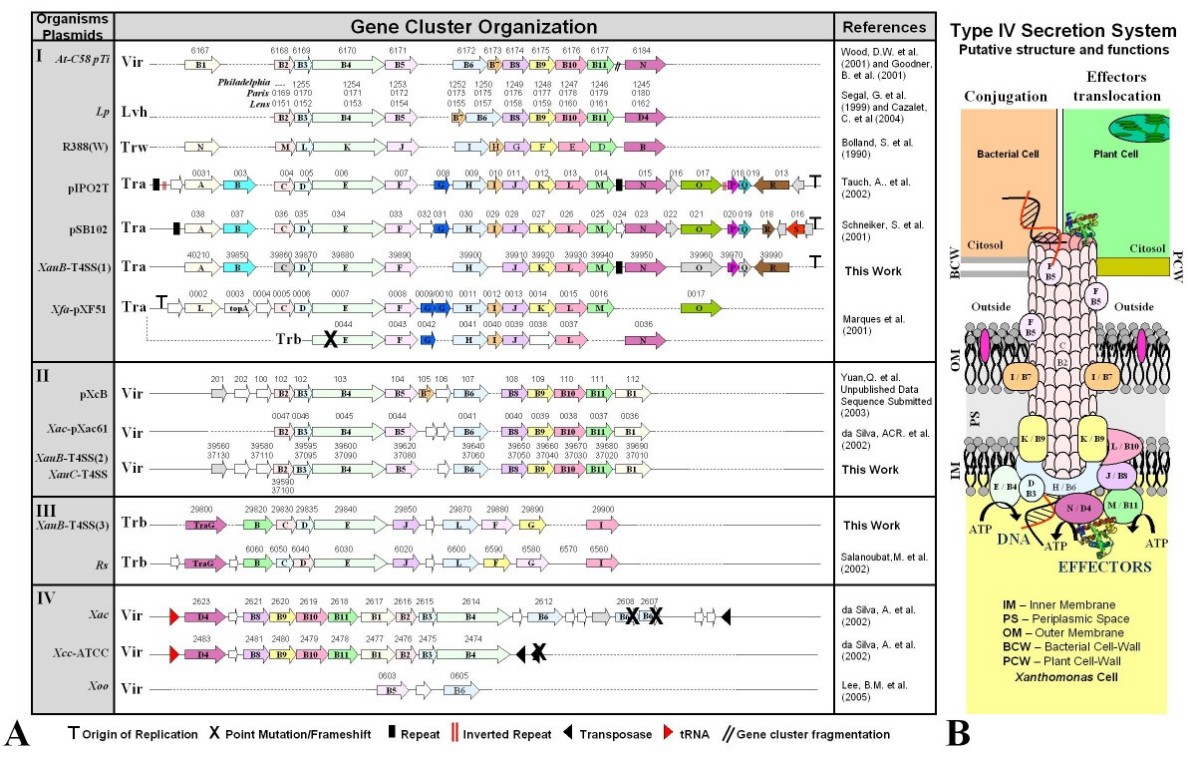
**Schematic representation of Type IV Secretion System Genes in diverse bacteria, grouped by their genomic architecture**. The colors used for gene arrows correspond to the colors used for their respective protein products in the secretion system representation on the right. *At: Agrobacterium tumefaciens *C58; *Lp: Legionella pneumophila*; *Xfa: Xylella fastidiosa *9a5c; *Rs: Ralstonia solanacearum*; Xcc*: Xanthomonas campestris *pv. *campestris *ATCC33913; *Xoo: Xanthomonas oryzae *pv. *oryzae*. References not cited in the text are: Goodner et al. (2001) [113]; Segal et al. (1999) [114]; Cazalet et al. (2004) [115]; Bolland et al. (1990) [116]; Marques et al. (2001) [117]; Lee et al. (2005) [118].

In addition to the type III secretion system, the type IV secretion system (T4SS) also plays a role in pathogenicity. For example, this system has been shown to contribute to full virulence in the phytopathogen *X. campestris *pv. *campestris *strain 8004 [88]. Genome clusters containing T4SS genes are found in several bacterial species but with marked differences in terms of gene presence/absence and organization, which relate to the system's function in the organism where it is present [89,90]. All three genomes under study contain T4SS genes, arrayed in clusters. In order to better understand these gene clusters we have classified the T4SS genes found in the three CC genomes as well as T4SS gene clusters from other organisms into four groups, using as criteria presence/absence of genes and synteny (Fig. 7). We have found important differences between XAC and XauB using this classification; data for XauC was too fragmented to allow a similar general observation.

As reported by da Silva et al. [12] XAC has two T4SS clusters, one in the chromosome and the other in a plasmid (pXAC64). In XauB we found three clusters. Only one of them is similar to a XAC cluster (the pXAC64 cluster), and was therefore placed in group II, along with the T4SS cluster found on plasmid pXcB (already mentioned above in the context of the *pth *gene discussion). The other two XauB clusters were placed in groups I and III, respectively, while the XAC chromosome cluster was placed in group IV (Fig. 7A).

The XauB cluster placed in group III is similar to a cluster found in *Ralstonia solanacearum *[91], both in terms of organization as well as individual gene sequence similarity. This organization is quite different from those found in other bacterial species. This XauB cluster is found in a region containing 45 genes, all of which are XauB-specific when compared to other *Xanthomonas *genomes. Moreover this cluster is flanked by insertion elements. This evidence suggests that this cluster was likely acquired by lateral transfer.

The third XauB cluster was placed in group I, which contains T4SS clusters similar to those found in *Agrobacterium tumefaciens *C58 plasmid Ti [92], *Xylella fastidiosa *9a5c plasmid pXF51 [93], and rhizosphere plasmids pIPO2T and pSB102 [94,95]. This XauB cluster is most similar to those found in the rhizosphere plasmids, in particular to pIPO2T. The XauC draft genome sequence did allow the identification of one T4SS gene cluster, and it belongs to this group (Fig. 7A).

**Figure 8:**
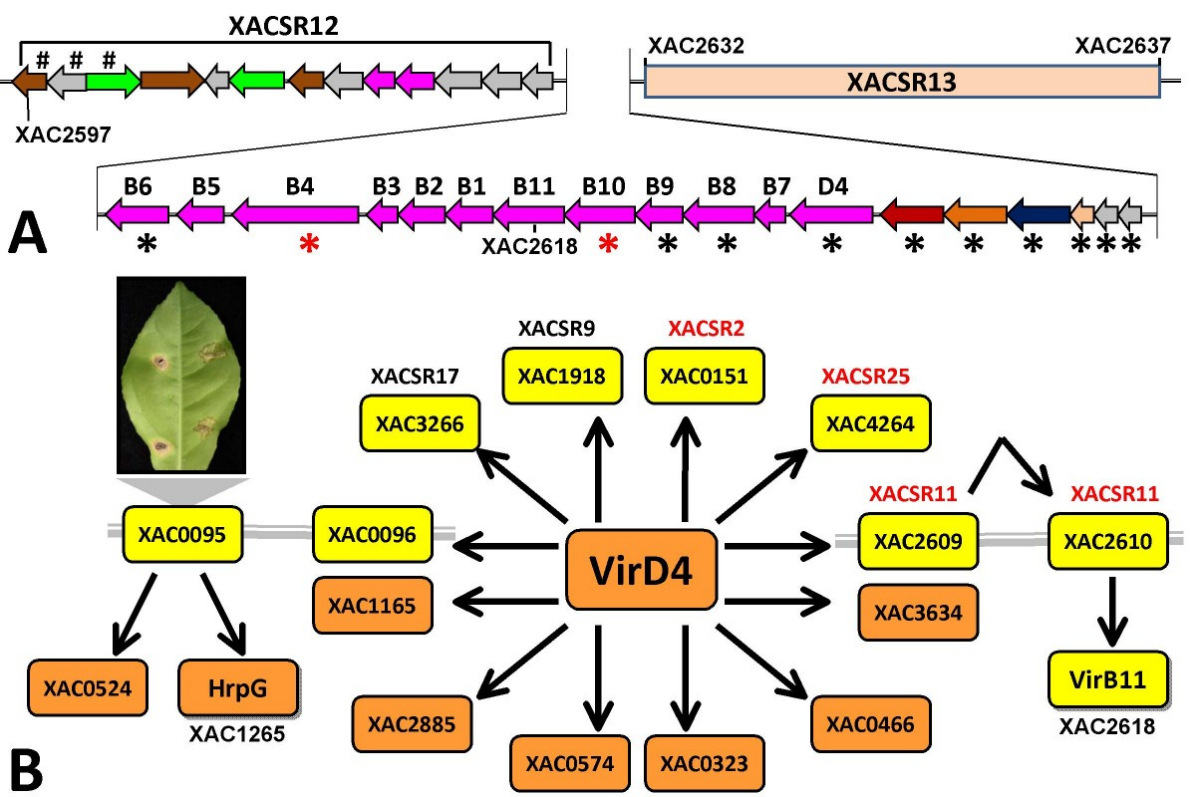
**The chromosomal T4SS gene cluster in XAC**. **(A) **This T4SS cluster is located between regions XACSR12 and XACSR13. Genes shared with XauB and XauC are shown with a black asterisk. Two of the genes are shared only by XAC and XauB (red asterisk). **(B) **Interactions among T4SS proteins, based on data presented by Alegria et al. [77]. Proteins specific to XAC are represented in yellow, and proteins shared by XAC, XauB and XauC are represented in orange. Six of the proteins colored in yellow are in XAC-specific regions. Genes XAC0095 and XAC0096, although not part of XAC-specific regions, seem to play an especially important role in pathogenicity. Under this model the protein coded for by XAC0095 interacts with HrpG, a protein that participates in the T3SS apparatus [41], and that causes phenotypic changes when its gene is mutated [31]. The product of gene XAC0096, which is next to XAC0095, interacts wirth VirD4, a component of the T4SS [77].

The XAC chromosomal cluster belongs to group IV (Fig. 7A). It is flanked by regions XACSR12 and XACSR13 (Fig. 8A). In addition, some of the genes in this T4SS cluster had uncertain hybridizations (zoomed-in portion of Fig. 5). This adds evidence that this cluster, as a specific unit, is indeed absent from both Xau genomes. XACSR12 contains genes that code for transporters, alpha-glucosidases, hypothetical genes and transposases, as well as a *virB6*-related gene. XACSR13 contains only hypothetical genes and transposases.

One of the members of this T4SS cluster is *virD4 *(XAC2623). Alegria et al. [77] have identified 12 XAC proteins that interact with VirD4. Six of these (XAC0096, 3266, 0151, 4264, 2609, 1918) are apparently absent from the Xau genomes, and five belong to XAC-specific regions (Fig. 8B). These genes might thus contribute to the increased virulence of XAC when compared to XauB and XauC.

### Flagellum and motility

**Figure 9:**
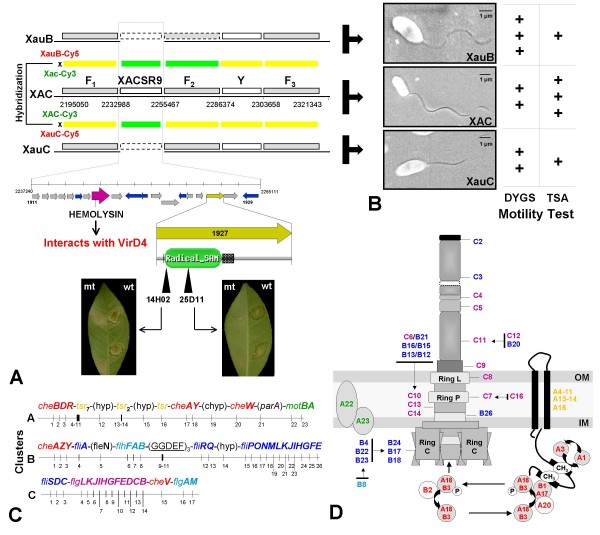
**Genes related to flagellum synthesis and regulation**. **(A) **Synteny and hybridization results between XAC e XauB/XauC. In the upper left diagram, F_1_, F_2 _and F_3 _denote gene regions related to flagellar functions, whereas gene groups XACSR9 and Y appear to contain unrelated genes. Dashed boxes denote regions apparently absent from the XauB and XauC genomes. Horizontal green bars represent hybridization signals for probes marked with Cy3 and which are XAC-specific; yellow horizontal bars represent regions with positive hybridization results. The zoomed-in region XACSR9 shows that it contains four transposases (in blue), twelve hypothetical genes (in gray), and one gene with assigned function (in yellow). That gene (XAC1927) codes for a Fe-S-oxidoreductase that contains a RADICAL SAM domain. XAC mutants in this particular region (14H02 and 25D11 [31]) presented decreased virulence phenotypes. **(B) **Microscopy and motility test of each organism. These results validate flagellum presence and functionality in two different culture media (DYGS and TSA). **(C) **Flaggellum gene organization in gene clusters F_1_, F_2 _and F_3_, as given in panel A. The color coding is the same for panel D. **(D) **Representation of the flagellum proteins using the same color coding as in panel C.

The XAC genome contains all genes known to be needed for flagellum synthesis [12-14]. Three of these clusters (which we call F_1_, F_2_, and F_3_) are close to the terminus of replication and are spread out over a 126 kbp region (Fig. 9A). A fourth cluster contains just two genes, coding for flagellar motor proteins A and B (*motA *and *motB*). All four clusters are found in both XauB and XauC with one exception: in XauB we did not find cluster F_2 _(XAC1930-XAC1955, 31 kbp), which contains 26 genes, coding for flagellum proteins that interact with bacterial membranes and coding for flagellar motor components (Fig. 9CD). On the other hand we have observed (microscopically) that XauB does have a flagellar structure similar to those of XAC and XauC, and motility tests have shown that XauB is able to move (Fig. 9B). Although our genome sequence data for XauB is incomplete, we believe it unlikely that a 31 kbp long fragment would be entirely missed; moreover, the hybridization experiment did not indicate presence of this fragment. These results suggest that the absence of genes in cluster F_2 _is compensated in XauB by other as yet undetermined genes.

We did not find in XauB nor in XauC genes that lie between clusters F_1 _and F_2_; this region is XACSR9. In this region the gene XAC1927 has been shown to be important for citrus canker since mutations in it significantly decreased virulence [31] (Fig. 9A).

### LPS and O-antigen genes

**Figure 10:**
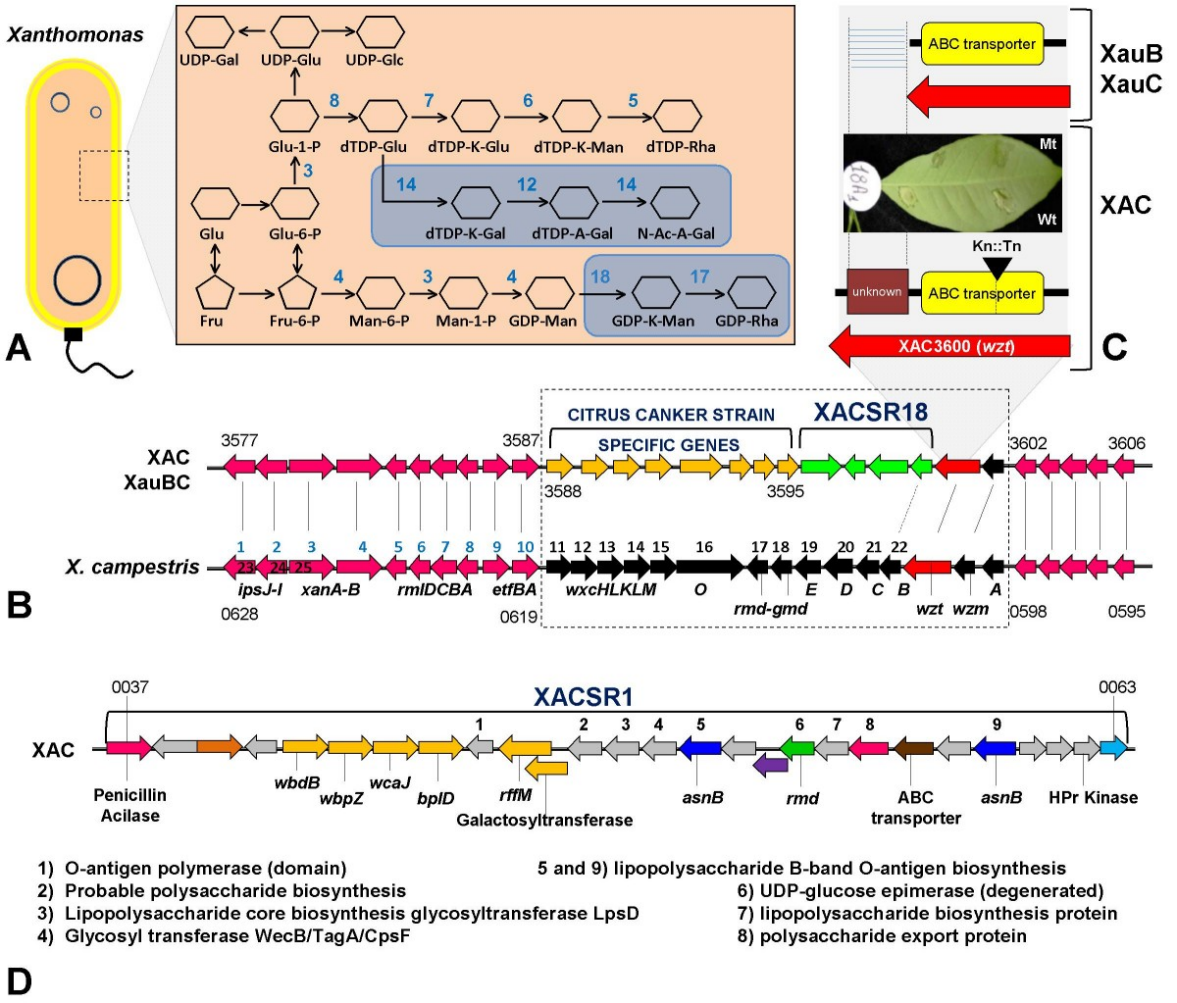
**LPS and O-antigen synthesis**. **(A) **Model representing LPS and O-antigen synthesis in *Xanthomonas*. Each of the numbers in this panel correspond to the same numbers in panel B. The model is based on the one described by Vorholter et al. [96] for *X. campestris*. Genes for products 12, 14, 17 and 18 are absent from XAC, XauB and XauC. **(B) **Genomic context of the LPS synthesis-related genes in XAC and *X. campestris*. The flanking regions (in pink) are conserved. The central region is not conserved between *X. campestris *on the one hand and XAC, XauB and XauC on the other. Moreover there is a group of genes that is shared and syntenic between the CC-species (in orange) and another (in green) that is specific to XAC (XACSR18). **(C) **A XAC mutant for gene *wzt *showed a different virulence pattern [31]. The *wzt *homolog in XauB and XauC does not have the C-terminal portion when compared to the XAC *wzt *gene. **(D) **Region XACSR1 contains several genes related to LPS biosynthesis. Numbers above gene arrows correspond to the annotations immediately below in this panel.

Two of the XAC-specific regions contain genes related to lipopolysaccharides (LPS) that may play a role in the differing phenotypes between XAC and the two Xau strains. Region XACSR18 (XAC3596-3599) (which contains gene *rfbC*, a truncated O-antigen biosynthesis protein) is immediately downstream of a CC-specific region (XAC3588-3595) (Fig. 10). An alignment with *X. campestris *pv. *campestris *ATCC33913 shows that these two regions together occupy roughly the same genomic locus as a 25-gene region related to LPS synthesis in *X. campestris *pv. *campestris *[96] (Fig. 10). Immediately downstream of XACSR18 we find genes *wzt *(XAC3600) and *wzm *(XAC3601), also described as part of the LPS synthesis cluster in *X. campestris *[96]. Laia et al. [31] obtained significant phenotypic differences in XAC (less necrosis, more water soaking and more hyperplasia, as compared to the wild type) by mutating gene XAC3600 (which codes for an ABC transporter ATP-binding protein). The *wzt *gene is present in both Xau genomes (XAUB_16610 and XAUC_09380), although both genes have lost the C-terminus when compared to the XAC *wzt *gene (Fig. 10). The *wzm *gene is also present in both Xau genomes (XAUB_ 16600 and XAUC_09370).

XACSR1 (XAC0037-0063) contains several genes related to LPS synthesis, including two copies of *asnB *(XAC0051 and XAC0059), which codes for an asparagine synthase. A homolog of this gene in *Pseudomonas aeruginosa *has been implicated in O-antigen biosynthesis [97].

### The gene xacPNP

Gottig et al. [98] have identified a plant natriuretic peptide-like protein in XAC (xacPNP), encoded by gene XAC2654. They have shown that a XAC2654-deletion mutant resulted in more necrotic tissues and earlier bacterial cell death than in the wild type. XAC2654 lies between regions XACSR13 and XACSR14, and is flanked on both sides by phage-related genes. We have experimentally verified that neither XauB nor XauC appears to contain a homolog of XAC2654 (Additional file 5: Fig. S5). Therefore, *xacPNP *might be another gene contributing to the higher virulence displayed by XAC as compared to XauB and XauC.

## Conclusions

Citrus canker continues to be an economically important disease. The publication of the XAC strain 306 genome in 2002 opened up new avenues of research, and several important insights into the genetics of canker have been obtained since then, most of them cited here. Yet, understanding the genomic basis of a bacterial plant disease is a complex undertaking. By obtaining the genome sequences of two additional citrus canker strains we have uncovered several new clues towards a thorough understanding of this disease.

We have approached the problem from two basic perspectives. The first was to determine commonalities among the three CC strains that were not found in other *Xanthomonas *genomes. Such traits are excellent candidates for the general genomic basis of canker and/or adaptation to citrus hosts. The second was to carefully compare the three CC genomes to one another, with special attention to genes that XAC has that the others (apparently) do not, as well as genes present in the Xau genomes but absent in XAC. Because of the draft nature of the Xau genomes here presented, all results concerning gene absence in their sequences are tentative. However, our hybridization platform provided additional evidence for the specificity of XAC genes and regions with respect to the other two strains.

Our most important findings are related to presence/absence of effector genes. In addition to the already known *pthA *gene, the genes *xopE3 *and *xopAI *deserve special attention in future studies. Moreover, we have identified several genes (such as *xacPNP*) that differentiate XAC from XauB and/or XauC. These genes or their homologs in other bacterial plant pathogens have demonstrated roles in virulence and/or host specificity. Hypotheses on their role in citrus canker and in host range differences between CC strains can now be tested experimentally.

We anticipate that knowledge in regard to CC-specific effectors and other CC-specific genes will be used in the future to engineer citrus species with durable resistance to citrus canker, thus reducing the economic impact of this disease on the citrus industry worldwide. Such knowledge will also be crucial for dealing with new canker variants that may emerge in the field, exemplified by the recent detection of what appears to be a new variant of *Xanthomonas fuscans *subsp. *aurantifolii *in swingle citrumelo [24].

## Methods

### Bacterial strains and DNA sequencing

The *Xanthomonas fuscans *subsp. *aurantifolii *type B genome sequenced was strain 11122 (B-69), isolated from a *Citrus limon *tree in Argentina. The *Xanthomonas fuscans *subsp. *aurantifolii *type C genome sequenced was strain 10535 (IBSBF338), isolated from a Mexican lime tree in São Paulo state in Brazil. We sequenced the genomes using the Sanger technique as described previously [12], with sequencers ABI 3700 and ABI 3100. For XauB we generated both shotgun and cosmid libraries; for XauC only shotgun libraries were created. For XauB we obtained 114,874 reads; for XauC we obtained 114,805 reads. We estimate this provided about 15× average coverage for each genome.

### Microscopy and motility tests

Initial attempts to visualize the flagellum of the two Xau strains with a scanning electron microscope by the normal routine (adhesion of the cultured bacteria on a cover slip with the help of the cationic compound poly-L-lysine, fixation, dehydration and critical point drying) failed because apparently attachment of the flagellum is very weak and it tends to fall off easily. To circumvent the problem, a diluted suspension of the bacterial culture was directly transferred onto a cover slip and excess of liquid eliminated. A moist chamber was made in a Petri dish where the cover slip was inserted together with a small plastic vial containing about 1 ml of 2% aqueous osmium tetroxide. The Petri dish was sealed and wrapped with aluminium foil and left overnight. The next morning the cover slip was removed, air dried and sputter-coated with gold, mounted on the stub and examined in a LEO 435 VP scanning electron microscope. Results are shown in Fig. 9B.

For motility tests TSA and DYGS media were used [27]. The Agar concentration of both media was changed (0.7%) so that the media were semisolid. For better growth visualization a phenol dye of 1% was added. Strains were placed in plates with solid media (TSA and DYGS) where isolated colonies were grown. The colonies were then placed in semisolid media. After 96 hours of incubation at 28°C, bacterial growth was observed in the test tubes, and results are given in the table in Fig. 9B.

### Gum production determination

Strains were maintained both in autoclaved tap water at room temperature and at -80°C in NA medium (3 g/l meat extract and 5 g/l peptone) containing 25% glycerol. The three strains were picked from -80°C stock, streaked on solid TSA medium (10 g/l tryptone, 10 g/l sucrose, 1 g/l sodium glutamate, and 15 g/l agar) and grown overnight at 28°C. One single isolated colony from each strain was streaked again on solid TSA medium and grown overnight at 28°C. For XAC, a single colony was inoculated into 20 ml of liquid TSA medium (10 g/l tryptone, 10 g/l sucrose, 1 g/l sodium glutamate) in a 125 ml Erlenmeyer flask and incubated at 28°C in a rotary shaker at 180 rpm for 17 hours (1.100 OD at 600 nm). A 125 ml Erlenmeyer flask containing 50 ml of liquid TSA medium was inoculated with 1 ml of XAC culture and incubated at 28°C in a rotary shaker at 180 rpm for 5 hours (0.300 OD at 600 nm). This XAC culture was used as inoculum for xanthan gum production. For XauB and XauC, an inoculating loop was used to inoculate the bacteria from the solid TSA medium plates into 20 ml of liquid TSA medium in a 50 ml Falcon tube, followed by incubation at 28°C in a rotary shaker at 180 rpm for 24.5 hours (0.260 and 0.550 OD at 600 nm for XauB and XauC, respectively). One ml of XauB and XauC culture was inoculated into separate 125 ml Erlenmeyer flasks containing 50 ml of liquid TSA medium and incubated at 28°C in a rotary shaker at 180 rpm for 15 hours (0.300 OD at 600 nm). The XauB and XauC cultures were used as inoculum for xanthan gum production.

For gum production, 2.5 ml of each bacterial strain in liquid TSA medium (0.300 OD at 600 nm) was inoculated in three (triplicate) 250 ml Erlenmeyer flasks containing 100 ml of media for xanthan gum production (25 g/l glucose, 3 g/l yeast extract, 2 g/l K_2_HPO_4_, 0.1 g/l MgSO_4_.7H_2_O, pH 7.0 with 4 M HCl [92]) and incubated at 28°C in a rotary shaker at 178 rpm for 96 h. The cells from the 96 h culture were centrifuged at 9.666 g for 40 min. The bacterial pellets were stored at -20°C and the supernatants were transferred to 500 ml beakers. The gum was recovered from the supernatants by alcohol precipitation. Four grams of KCl were added to each beaker followed by agitation at room temperature for 15 min. Two volumes of cold isopropyl alcohol were added and the gum from each beaker was removed to pre-weighted plastic discs. After 16 h at 37°C the discs were weighted again and the gum amount was calculated. The bacterial pellet from each culture was also obtained. For this, each pellet was transferred to a pre-weighted beaker and weighted again after 14 h at 70°C.

### DNA Microarray

From the shotgun libraries made for the sequencing of XAC strain 306 in 2001, 2,653 clones were selected for the design of a glass slide hybridization array. Inserts from selected clones were amplified by PCR with the M13-R and M13-F universal oligonucleotides. Products were purified and placed in duplicate slides, resulting in 6,144 probes, with 768 positive controls and 624 negative controls. Experimental validation was done by hybridization of total XAC DNA probes differentially stained, with 86% of all products with a hybridization signal greater than the average value for the noise signal plus two standard deviations. The final XACarray contains targets for the identification of 2,760 putative coding sequences (61% of all annotated protein-coding genes). The XACarray was used to compare XAC and XauC, and XAC and XauB, with XAC itself as a positive control. The results were similar (128 CDSs considered XAC-specific had similar ratios for both the XauB and XauC experiments, and 101 of these are in XACSRs); we report here detailed results only for the first experiment. Details of the array construction are described elsewhere (Moreira LM, Laia ML, de Souza RF, Zaini PA, da Silva ACR, Ferro JA, da Silva AM: Development and validation of a *Xanthomonas citri *subsp. *citri *DNA microarray platform (*XACarray*) generated from the shotgun libraries previously used in the sequencing of this bacterial genome, submitted).

### XacPNP verification

Evidence for the absence of genes coding for XacPNP homologs in XauB and XauC was obtained by PCR using gene-specific primers (Forward: GGACCAACAACGAATATC; Reverse: ATGGGAATAGTCATGAAAC). XAC was used as positive control.

### Assembly and genome annotation

Base calling, genome assembly and visualization were done with the phred-phrap-consed package [99-101]. Contigs were trimmed to remove low phred quality regions at both ends. For both XauB and XauC all contigs larger than 1 kbp have average phred quality greater than 20, and nearly all (99% for XauB and 96% for XauC) these contigs have average phred quality greater than or equal to 40 (i.e. accuracy equal to or better than 1 error in 10,000 bp). To improve assembly many transposase sequences were masked, and reads were re-assembled to obtain the final result. We estimated the fraction obtained of the complete genome of XauB by dividing the total length of contigs by the total genome size of XAC, and by adding 1% because of the removed transposases. We did the same with XauC. To estimate the average gap size we divided the number of contigs by the estimated size of un-sequenced genome.

Using paired-end reads scaffolds for the XauB and XauC chromosomes were obtained. The overall correctness of each scaffold was validated using a sliding-window GC-skew computation. Each scaffold was aligned against the XAC chromosome using MUMmer [102]. Results are presented in additional file 1 (Fig. S1).

The XauB and XauC genomes were automatically annotated with the Genome Reverse Compiler [103], with a few manual refinements. Ortholog groups were built using OrthoMCL [104].

### XAC-specific regions

For determination of XAC-specific regions we relied on published data about putative genomic islands [14,31,105] and on AlienHunter results [40]. Islands whose genes were not found in the XauB and XauC genomes by BLAST [45] analysis and with differential hybridization signals in the XACarray were considered XAC-specific regions.

### Phylogeny reconstruction

#### Species tree

We used a supermatrix approach as in previous work [106]. Protein sequences of eleven *Xanthomonas *genomes (ingroup) and four *Xylella *genomes (outgroup) were clustered in 6,375 families using OrthoMCL [104]. We then selected families with one and only one representative from each of the ingroup genomes and at least one outgroup protein, resulting in 1,666 families. Their sequences were aligned using MUSCLE [107] and the resulting alignments were concatenated. Non-informative columns were removed using Gblocks [108], resulting in 596,246 positions. RAxML [109] with the PROTGAMMAWAGF model was used to build the final tree.

#### Pth tree

The same methodology as above was used, with the following differences. Representative *pth *nucleotide sequences from *Xanthomonas *species were retrieved from GenBank, and added to the set of XauB and XauC *pth *nucleotide sequences. A *pth *gene from *Ralstonia solanacearum *[GenBank: CAD1557.1] was used as an outgroup in a preliminary round of tree construction to ascertain root position.  The list of gene sequences used to build the tree is given in additional file 6 (Table S6). The Tandem Repeat Finder program [110] with parameters 2,7,7,80,10,50,500,1 was used to mask the internal repeats. The masked regions were removed and the resulting sequences were aligned with MUSCLE. A few manual adjustments to the multiple alignment were made before running Gblocks, which yielded 1,679 positions. RAxML with the GTRGAMMA model was used to build the final tree.  The bootstrap values obtained for the clade indicated by an asterisk in Fig. 3 are given in additional file 7 (Fig. S7).

### Effector analysis

The candidate T3SS effectors in the XauB and XauC genomes were identified using tBLASTn [45] analysis and Pfam domain [111] searches. For tBLASTn analysis, all known plant and animal pathogen effectors were used as query with an e-value threshold ≤ 0.00001. Pfam domains were searched for possible domains found in known effectors in the predicted set of ORFs of draft genome sequences. Candidate effectors were classified according to the nomenclature and classification scheme for effectors in xanthomonads recently described by White et al. [112].

### Database submission

The draft genome sequences of XauB and XauC are available at GenBank under accession numbers ACPX00000000 and ACPY00000000, respectively.

## Abbreviations

CC: citrus canker; CDS: coding sequence; ORF: Open Reading Frame; PCR: polymerase chain reaction; T3SS: Type III secretion system; T4SS: Type IV secretion system; XAC: *Xanthomonas citri *subsp. *citri*; XACSR: XAC-specific region; Xau: *Xanthomonas fuscans *subsp. *aurantifolii*; XauB: *Xanthomonas fuscans *subsp. *aurantifolii *strain B; XauC: *Xanthomonas fuscans *subsp. *aurantifolii *strain C.

## Authors' contributions

JAF and ACRS conceived the project and oversaw genomic sequencing. RPL provided the strains. JCS oversaw all bioinformatics work, including assembly, annotation and other specialized analyses. MYN, EHO, and LAD helped with genome assembly. LAD and EHO did computational analyses. RIDT maintained the computational infrastructure. LMM conceived the XACarray. LMM and ALJF carried out hybridization experiments. HAP and LASN did the motility tests. EWK did microscopy analysis. DFRJ did the xanthan gum production experiment. JAF oversaw final PCR experiments. APF ran PCR analyses. NFA helped with automated genome annotation and manual refinement, created ortholog families, and generated the GenBank files. NFA, SSA and JCS did the phylogenetic analyses. APF, AMS, HAP, FEM, JCB, JCFO, LMM, LASN, LRF, MLL, MITF, RFS, and RIDT helped with data analyses. DJN and BJS helped create an effector database. NP did the effector analysis. NP, JBJ, BAV, LMM, and JCS interpreted effector analysis. LMM created all figures except Figs. 2 and 3. MLL, LRF, and JRN provided preliminary sections of the manuscript. LMM, BAV, and JCS wrote the final manuscript. All authors approved the final manuscript.

## Supplementary Material

Additional file 1 Figure S1: whole chromosome alignments of XauB and XauC scaffolds against XAC. Click here for file

Additional file 2 Table S2: genes shared by XAC, XauB, and XauC but that are not found in other fully sequenced *Xanthomonas *and *Xylella *genomes. Click here for file

Additional file 3 Table S3: List of XAC-specific regions. Click here for file

Additional file 4 Figure S4: gene diagrams showing the contents of all 25 XAC-specific regions. Click here for file

Additional file 5 Figure S5: PCR results for gene *xacPNP*. Click here for file

Additional file 6 **Table S6: names, organisms, locus tags, and accession numbers for the nucleotide sequences used to build the *pth *gene phylogeny (Fig.**3**).** Click here for file

Additional file 7 **Figure S7: topology of clade in Fig.**3**with bootstrap values.** Click here for file
