# Supplementary material for: Novel insights into the genomic basis of citrus canker based on the genome sequences of two strains of Xanthomonas fuscans subsp. aurantifolii
Source: BMC Genomics. 2010 Apr 13;11:238. doi: 10.1186/1471-2164-11-238 (PMC2883993; doi:10.1186/1471-2164-11-238)
Supplement: Additional file 1 — Figure S1: whole chromosome alignments of XauB and XauC scaffolds against XAC. [file 1471-2164-11-238-S1.PPT]

## Slide 1
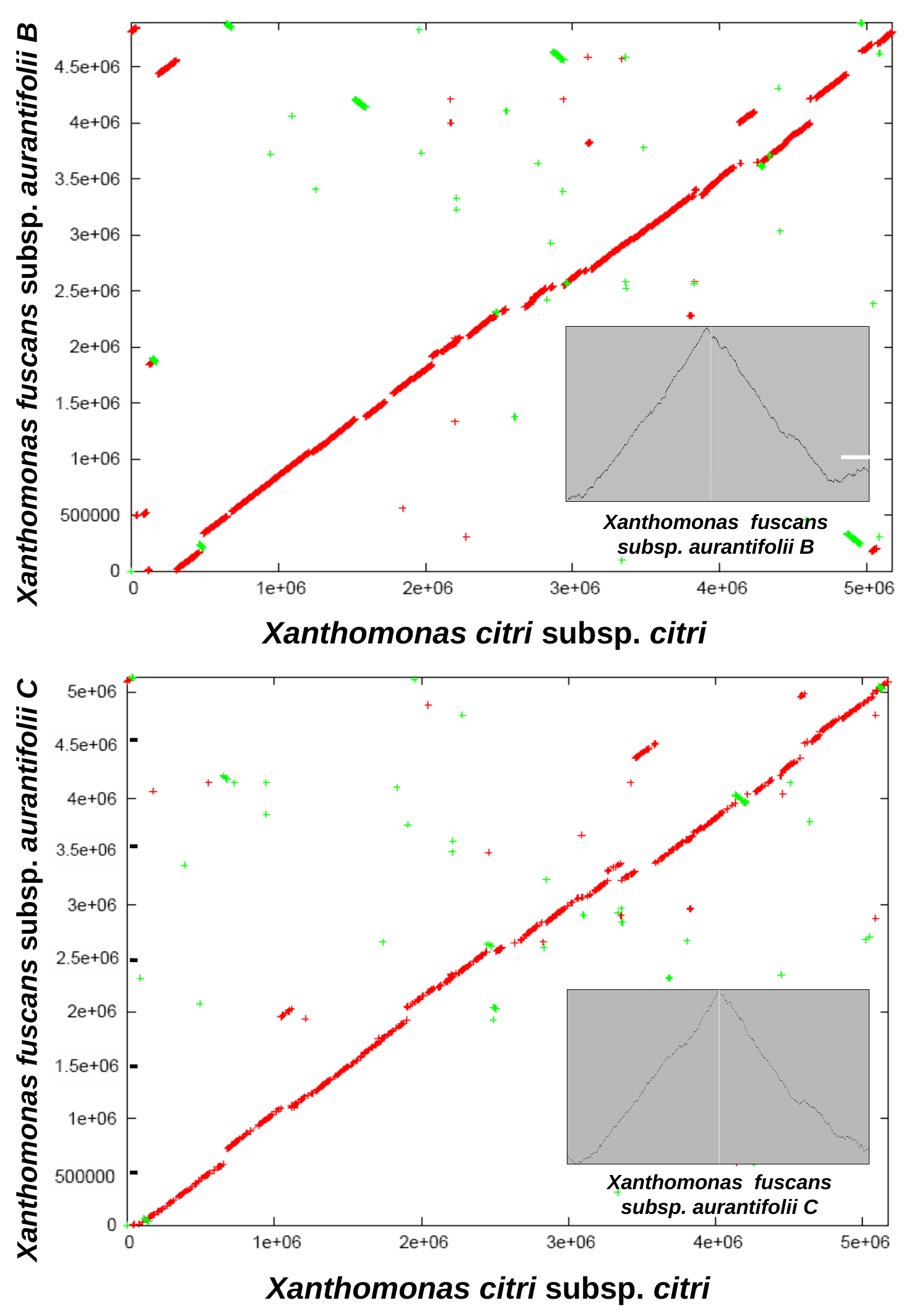

Xanthomonas fuscans subsp. aurantifolii B
Xanthomonas fuscans subsp. aurantifolii B
Xanthomonas citri subsp. citri
Xanthomonas fuscans subsp. aurantifolii C
Xanthomonas fuscans subsp. aurantifolii C
Xanthomonas citri subsp. citri
