# Supplementary material for: Novel insights into the genomic basis of citrus canker based on the genome sequences of two strains of Xanthomonas fuscans subsp. aurantifolii
Source: BMC Genomics. 2010 Apr 13;11:238. doi: 10.1186/1471-2164-11-238 (PMC2883993; doi:10.1186/1471-2164-11-238)
Supplement: Additional file 2 — Table S2: genes shared by XAC, XauB, and XauC but that are not found in other fully sequenced Xanthomonas and Xylella genomes. [file 1471-2164-11-238-S2.DOC]

**Table S2**. **Genes shared by XAC, XauB, and XauC but that are not found in other fully sequenced *Xanthomonas* and *Xylella* genomes**. Families were obtained with OrthoMCL (Li, Stoeckert, and Roos, *Genome Research*, 2003, 13:2178-2189). The asterisk * indicates that a nearby gene when mutated caused a phenotype with reduced virulence (Laia et al., *BMC Microbiology*, 2009, 9(12)). Syntenic blocks are indicated by shaded rows. Each such block is in a different color. Anomalous regions were determined with AlienHunter results (Vernikos and Parkhill, *Bioinformatics*, 2006 22:2196-2203) on the XAC chromosome.

| **Family** | **XAC** | | | | **XauB**  **Locus_tag** | **XauC**  **Locus_tag** | **Function assignment**  **(XAC annotation)** | **Anomalous**  **region** |
| --- | --- | --- | --- | --- | --- | --- | --- | --- |
|  | **ID** | **Strain** | **Start pos.** | **End pos.** |  |  |  |  |
| 4541 | XAC0085 |  | 103084 | 104085 | XAUB_16700 | XAUC_23720 | Conserved hypothetical protein | YES |
| 4540 | XAC0086 |  | 104350 | 104865 | XAUB_16690 | XAUC_23730 | Conserved hypothetical protein | YES |
| 4538 | XAC0134 |  | 157496 | 158680 | XAUB_16030 | XAUC_12950 | Beta-lactamase | NO |
| 4537 | XAC0283 |  | 336818 | 337519 | XAUB_36980 | XAUC_37550 | Hydrolase | NO |
| 4536 | XAC0291 |  | 344924 | 347917 | XAUB_37060 | XAUC_37630 | Oar protein | NO |
| 4535 | XAC0527 |  | 617360 | 617854 | XAUB_33570 | XAUC_15720 | Conserved hypothetical protein | NO |
| 4534 | XAC0599 |  | 702983 | 703486 | XAUB_23120 | XAUC_21280 | Conserved hypothetical protein | NO |
| 4532 | XAC0726 |  | 865373 | 866395 | XAUB_38810 | XAUC_30830 | Methyl parathion hydrolase | NO |
| 4531 | XAC0727 |  | 866458 | 867252 | XAUB_38820 | XAUC_30840 | Transcriptional regulator | NO |
| 4530 | XAC0845 |  | 1004256 | 1005383 | XAUB_21650 | XAUC_17170 | Transcriptional regulator | NO |
| 4529 | XAC0846 |  | 1005433 | 1006620 | XAUB_21640 | XAUC_17180 | FMNH2-dependent monooxygenase | NO |
| 4528 | XAC0847 |  | 1006745 | 1007503 | XAUB_21630 | XAUC_17190 | ABC transporter ATP-binding protein | NO |
| 4527 | XAC0848 |  | 1007500 | 1008351 | XAUB_21620 | XAUC_17200 | ABC transporter permease | NO |
| 4526 | XAC0849 |  | 1008360 | 1009364 | XAUB_21610 | XAUC_17210 | Sulfonate binding protein | NO |
| 4525 | XAC0850 |  | 1009376 | 1010539 | XAUB_21600 | XAUC_17220 | Alkanesulfonate monooxygenase | NO |
| 4524 | XAC0851 |  | 1010750 | 1011313 | XAUB_21590 | XAUC_17230 | NADH-dependent FMN reductase | NO |
| 4523 | XAC0852 |  | 1011721 | 1013925 | XAUB_21580 | XAUC_09010 | TonB-dependent receptor | NO |
| 4522 | XAC0854 |  | 1014835 | 1016139 | XAUB_03080 | XAUC_09000 | Conserved hypothetical protein | NO |
| 4521 | XAC0855 |  | 1016136 | 1017596 | XAUB_03070 | XAUC_08990 | Monooxygenase | NO |
| 4520 | XAC0856 |  | 1017611 | 1019227 | XAUB_03060 | XAUC_08980 | ABC transporter binding protein | NO |
| 4519 | XAC0858 |  | 1020198 | 1021082 | XAUB_18530 | XAUC_08960 | ABC transporter permease | NO |
| 4518 | XAC0860 |  | 1021286 | 1022728 | XAUB_18540 | XAUC_08950 | ABC transporter ATP-binding protein | NO |
| 4516 | XAC1388 |  | 1595393 | 1598989 | XAUB_20390 | XAUC_10530 | Conserved hypothetical protein | NO |
| 4515 | XAC1389 |  | 1599002 | 1599895 | XAUB_20400 | XAUC_10520 | ABC transporter ATP-binding protein | NO |
| 4512 | XAC1613 |  | 1859901 | 1860470 | XAUB_24450 | XAUC_12090 | Conserved hypothetical protein | NO |
| 4511 | XAC1614 |  | 1860674 | 1861363 | XAUB_24460 | XAUC_19550 | Conserved hypothetical protein | NO |
| 4509 | XAC2119* |  | 2476483 | 2477103 | XAUB_14670 | XAUC_07620 | Conserved hypothetical protein | NO |
| 4500 | XAC2673 |  | 3133802 | 3134287 | XAUB_08600 | XAUC_12210 | Conserved hypothetical protein | NO |
| 4499 | XAC2860 |  | 3355194 | 3357104 | XAUB_06330 | XAUC_19060 | Conserved hypothetical protein | NO |
| 4498 | XAC2862 |  | 3358551 | 3359192 | XAUB_03690 | XAUC_43420 | Conserved hypothetical protein | NO |
| 4497 | XAC3050 |  | 3567839 | 3570760 | XAUB_33790 | XAUC_34090 | TonB-dependent receptor | YES |
| 4428 | XAC3077 |  | 3607286 | 3610177 | XAUB_34020 | XAUC_12600 | TonB-dependent receptor | NO |
| 4496 | XAC3079 |  | 3612182 | 3613549 | XAUB_34030 | XAUC_12580 | Cationic amino acid transporter | NO |
| 4495 | XAC3080 |  | 3613528 | 3614463 | XAUB_34040 | XAUC_12570 | Ribokinase | NO |
| 4494 | XAC3081 |  | 3614460 | 3615884 | XAUB_34050 | XAUC_12560 | 6-phospho-beta-glucosidase | NO |
| 4493 | XAC3088 |  | 3625907 | 3626212 | XAUB_34110 | XAUC_12500 | Conserved hypothetical protein | NO |
| 4492 | XAC3131 |  | 3682724 | 3683038 | XAUB_29300 | XAUC_14480 | Conserved hypothetical protein | NO |
| 4491 | XAC3183 |  | 3746178 | 3747353 | XAUB_16920 | XAUC_01580 | Conserved hypothetical protein | NO |
| 4490 | XAC3197 |  | 3762283 | 3763077 | XAUB_10950 | XAUC_29340 | ABC transporter permease | NO |
| 4489 | XAC3198 |  | 3763196 | 3764221 | XAUB_10940 | XAUC_29350 | Alkanesulfonate transporter substrate binding | NO |
| 4488 | XAC3199 |  | 3764320 | 3765360 | XAUB_10930 | XAUC_29360 | oxidoreductase | NO |
| 4487 | XAC3200 |  | 3765377 | 3766705 | XAUB_10920 | XAUC_29370 | Nitrilotriacetate monooxygenase component A | NO |
| 4486 | XAC3224* |  | 3797831 | 3798901 | XAUB_14680 | XAUC_00040 | Avirulence protein (type III SS XopE3) | NO |
| 4485 | XAC3230 |  | 3806072 | 3806962 | XAUB_26830 | XAUC_23780 | Avirulence protein (type III SS XopAI) | YES |
| 4484 | XAC3233 |  | 3808805 | 3809920 | XAUB_03570 | XAUC_00450 | Transposase | YES |
| 4483 | XAC3234 |  | 3809875 | 3810390 | XAUB_20110 | XAUC_00440 | Conserved hypothetical protein | YES |
| 4482 | XAC3270 |  | 3851918 | 3852886 | XAUB_29760 | XAUC_00380 | Conserved hypothetical protein | YES |
| 4477 | XAC3337 |  | 3933209 | 3933577 | XAUB_15050 | XAUC_30350 | Conserved hypothetical protein | NO |
| 4475 | XAC3588 |  | 4254271 | 4255149 | XAUB_05370 | XAUC_12710 | Integral membrane protein | BORDER |
| 4426 | XAC3589 |  | 4255252 | 4256640 | XAUB_05360 | XAUC_12700 | Conserved hypothetical protein | BORDER |
| 4025 | XAC3590 |  | 4256640 | 4257941 | XAUB_05350 | XAUC_12680 | Oxidoreductase | BORDER |
| 4474 | XAC3591 |  | 4257944 | 4258672 | XAUB_05340 | XAUC_12670 | Short chain dehydrogenase | BORDER |
| 4473 | XAC3593 |  | 4261220 | 4262164 | XAUB_05330 | XAUC_12660 | NAD dependent epimer./dehydrat./dehydrogen. | BORDER |
| 4472 | XAC3594 |  | 4262187 | 4263473 | XAUB_02700 | XAUC_12650 | Conserved hypothetical protein | YES |
| 4471 | XAC3595* |  | 4263470 | 4263868 | XAUB_02690 | XAUC_12640 | Conserved hypothetical protein | YES |
| 4470 | XAC3611 |  | 4283663 | 4285507 | XAUB_16490 | XAUC_04090 | Peptidase | NO |
| 4469 | XAC3619 |  | 4291954 | 4292916 | XAUB_11810 | XAUC_06250 | Conserved hypothetical protein | NO |
| 4468 | XAC3620 |  | 4292976 | 4295219 | XAUB_11820 | XAUC_06260 | Outer membrane receptor FepA | NO |
| 4467 | XAC3767 |  | 4443316 | 4443744 | XAUB_02890 | XAUC_42890 | Conserved hypothetical protein | NO |
| 4466 | XAC3874 |  | 4557246 | 4557638 | XAUB_25780 | XAUC_27320 | Conserved hypothetical protein | NO |
| 4465 | XAC3968 |  | 4665433 | 4665855 | XAUB_28410 | XAUC_07270 | Conserved hypothetical protein | NO |
| 4463 | XAC4104 |  | 4806727 | 4807071 | XAUB_28690 | XAUC_17760 | Conserved hypothetical protein | NO |
| 4458 | XAC4253 |  | 5020301 | 5020702 | XAUB_19700 | XAUC_34520 | Conserved hypothetical protein | NO |
| 4457 | XAC4326 |  | 5111104 | 5114751 | XAUB_03730 | XAUC_14760 | Urea amidolyas | NO |
| 4456 | XAC4327 |  | 5114835 | 5116634 | XAUB_10310 | XAUC_14750 | Amidase | NO |
| 4452 | XACa0041 |  |  |  | 99828500 | 99922440 | Partition protein A | NO |
