# Supplementary material for: Novel insights into the genomic basis of citrus canker based on the genome sequences of two strains of Xanthomonas fuscans subsp. aurantifolii
Source: BMC Genomics. 2010 Apr 13;11:238. doi: 10.1186/1471-2164-11-238 (PMC2883993; doi:10.1186/1471-2164-11-238)
Supplement: Additional file 3 — Table S3: List of XAC-specific regions. [file 1471-2164-11-238-S3.DOC]

**Table S3.** **List of XAC-specific regions**. XACSR – Region Number; Start LT: locus tag of first protein-coding gene in the region; End LT: locus tag of last protein-coding gene in the region; Start Pos.: left border of region (nucleotide position); End Pos.: right border of region (nucleotide position); NG – total number of protein coding genes in region; NGS – number of XAC-specific genes (percent of NG); NGM – number of mobile elements; NGH – number of genes without functional assignment; TGR – number of genes represented in plate (percent of NG); TGRR>2 – number of genes in region with hybridization ratio greater than 2 (percent of TGR).

| ***XAC***  ***SR*** | ***Genetic composition of the Regions*** | | | | | | | | ***tRNA*** | | | ***Transposase*** | | ***Integrase*** | | ***XACarray*** | |
| --- | --- | --- | --- | --- | --- | --- | --- | --- | --- | --- | --- | --- | --- | --- | --- | --- | --- |
|  | ***Start***  ***LT*** | ***End***  ***LT*** | ***Start***  ***Pos.*** | ***End***  ***Pos.*** | ***NG*** | ***NGS***  ***(%)*** | ***NGM*** | ***NGH*** | | ***5’*** | ***3’*** | ***5’*** | ***3’*** | ***5’*** | ***3´*** | ***TGR***  ***(%)*** | ***TGRR>2***  ***(%)*** |
| ***1*** | ***0037*** | ***0063*** | ***46368*** | ***77539*** | ***27*** | ***23 (85)*** | ***0*** | ***12*** | ***----*** | | ***----*** | ***No*** | ***No*** | ***No*** | ***No*** | ***19 (70)*** | ***6 (32)*** |
| ***2*** | ***0146*** | ***0152*** | ***176973*** | ***184553*** | ***7*** | ***6 (85)*** | ***3*** | ***4*** | ***----*** | | ***----*** | ***No*** | ***Yes*** | ***No*** | ***No*** | ***5 (71)*** | ***3 (60)*** |
| ***3*** | ***1051*** | ***1072*** | ***1209505*** | ***1228677*** | ***22*** | ***20 (91)*** | ***10*** | ***11*** | ***PRH*** | | ***KL*** | ***Yes*** | ***Yes*** | ***No*** | ***No*** | ***15 (68)*** | ***7 (47)*** |
| ***4*** | ***1101*** | ***1107*** | ***1253185*** | ***1259423*** | ***7*** | ***5 (71)*** | ***4*** | ***2*** | ***----*** | | ***S*** | ***Yes*** | ***No*** | ***No*** | ***Yes*** | ***7 (100)*** | ***1 (14)*** |
| ***5*** | ***1491*** | ***1509*** | ***1723743*** | ***1744015*** | ***20*** | ***9 (45)*** | ***6*** | ***13*** | ***----*** | | ***----*** | ***No*** | ***No*** | ***No*** | ***Yes*** | ***12 (60)*** | ***4 (30)*** |
| ***6*** | ***1657*** | ***1664*** | ***1908975*** | ***1919214*** | ***8*** | ***6 (75)*** | ***4*** | ***4*** | ***S*** | | ***----*** | ***No*** | ***No*** | ***No*** | ***Yes*** | ***6 (75)*** | ***3 (50)*** |
| ***7*** | ***1810*** | ***1819*** | ***2083535*** | ***2113580*** | ***10*** | ***8 (80)*** | ***0*** | ***2*** | ***R*** | | ***----*** | ***No*** | ***No*** | ***No*** | ***No*** | ***6 (60)*** | ***0 (00*** |
| ***8*** | ***1866*** | ***1872*** | ***2164332*** | ***2172365*** | ***7*** | ***5 (71)*** | ***2*** | ***4*** | ***----*** | | ***----*** | ***No*** | ***Yes*** | ***No*** | ***No*** | ***5 (71)*** | ***1 (20)*** |
| ***9*** | ***1911*** | ***1929*** | ***2237340*** | ***2255111*** | ***19*** | ***15 (79)*** | ***4*** | ***5*** | ***----*** | | ***----*** | ***Yes*** | ***Yes*** | ***No*** | ***No*** | ***14 (74)*** | ***4 (29)*** |
| ***10*** | ***2174*** | ***2286*** | ***2547786*** | ***2675160*** | ***113*** | ***96 (84)*** | ***17*** | ***58*** | ***----*** | | ***----*** | ***Yes*** | ***No*** | ***No*** | ***Yes*** | ***70 (61)*** | ***35 (50)*** |
| ***11*** | ***2418*** | ***2445*** | ***2818642*** | ***2847010*** | ***28*** | ***16 (57)*** | ***12*** | ***13*** | ***A*** | | ***----*** | ***Yes*** | ***No*** | ***No*** | ***No*** | ***19 (68)*** | ***10 (53)*** |
| ***12*** | ***2601*** | ***2609*** | ***3068262*** | ***3075701*** | ***11*** | ***9 (69)*** | ***2*** | ***6*** | ***----*** | | ***V*** | ***Yes*** | ***No*** | ***No*** | ***No*** | ***9 (69)*** | ***8 (89)*** |
| ***13*** | ***2632*** | ***2637*** | ***3096409*** | ***3103662*** | ***6*** | ***5 (83)*** | ***2*** | ***4*** | ***----*** | | ***----*** | ***No*** | ***No*** | ***Yes*** | ***Yes*** | ***4 (67)*** | ***0 (0)*** |
| ***14*** | ***2660*** | ***2673*** | ***3119404*** | ***3134287*** | ***14*** | ***7 (50)*** | ***4*** | ***3*** | ***----*** | | ***----*** | ***Yes*** | ***No*** | ***No*** | ***No*** | ***10 (71)*** | ***2 (20)*** |
| ***15*** | ***2901*** | ***2904*** | ***3406101*** | ***3411288*** | ***4*** | ***4 (100)*** | ***1*** | ***3*** | ***----*** | | ***----*** | ***No*** | ***No*** | ***No*** | ***Yes*** | ***4 (100)*** | ***0 (0)*** |
| ***16*** | ***3014*** | ***3025*** | ***3524613*** | ***3532867*** | ***12*** | ***12 (100)*** | ***0*** | ***8*** | ***----*** | | ***----*** | ***No*** | ***No*** | ***No*** | ***No*** | ***9 (75)*** | ***2 (22)*** |
| ***17*** | ***3245*** | ***3298*** | ***3823370*** | ***3877600*** | ***54*** | ***36 (67)*** | ***7*** | ***29*** | ***----*** | | ***G*** | ***No*** | ***Yes*** | ***No*** | ***Yes*** | ***43 (81)*** | ***9 (21)*** |
| ***18*** | ***3596*** | ***3599*** | **4263865** | ***4270247*** | ***4*** | ***3 (75)*** | ***0*** | ***1*** | ***----*** | | ***----*** | ***No*** | ***No*** | ***No*** | ***No*** | ***3 (75)*** | ***2 (66)*** |
| ***19*** | ***3770*** | ***3785*** | ***4447486*** | ***4454908*** | ***16*** | ***14 (87)*** | ***1*** | ***12*** | ***----*** | | ***M*** | ***No*** | ***No*** | ***No*** | ***Yes*** | ***5 (63)*** | ***1 (20)*** |
| ***20*** | ***3836*** | ***3843*** | ***4512469*** | ***4522281*** | ***8*** | ***8 (100)*** | ***0*** | ***8*** | ***----*** | | ***----*** | ***No*** | ***No*** | ***No*** | ***No*** | ***5 (31)*** | ***1 (20)*** |
| ***21*** | ***3932*** | ***3955*** | ***4624144*** | ***4651896*** | ***24*** | ***16 (66)*** | ***5*** | ***14*** | ***----*** | | ***----*** | ***No*** | ***No*** | ***Yes*** | ***No*** | ***12 (50)*** | ***4 (33)*** |
| ***22*** | ***4061*** | ***4064*** | ***4756620*** | ***4761806*** | ***4*** | ***4 (100)*** | ***0*** | ***3*** | ***----*** | | ***----*** | ***No*** | ***No*** | ***No*** | ***No*** | ***4 (100)*** | ***2 (50)*** |
| ***23*** | ***4135*** | ***4139*** | ***4858635*** | ***4865544*** | ***5*** | ***5 (100)*** | ***2*** | ***3*** | ***----*** | | ***----*** | ***No*** | ***Yes*** | ***No*** | ***No*** | ***2 (50)*** | ***0 (0)*** |
| ***24*** | ***4261*** | ***4266*** | ***5030510*** | ***5035413*** | ***6*** | ***6 (100)*** | ***0*** | ***6*** | ***----*** | | ***----*** | ***No*** | ***No*** | ***No*** | ***No*** | ***3 (50)*** | ***2 (66)*** |
| ***25*** | ***4314*** | ***4338*** | ***5099881*** | ***5134021*** | ***25*** | ***19 (76)*** | ***4*** | ***12*** | ***----*** | | ***----*** | ***No*** | ***No*** | ***No*** | ***No*** | ***14 (56)*** | ***1 (7)*** |
