# Supplementary material for: Novel insights into the genomic basis of citrus canker based on the genome sequences of two strains of Xanthomonas fuscans subsp. aurantifolii
Source: BMC Genomics. 2010 Apr 13;11:238. doi: 10.1186/1471-2164-11-238 (PMC2883993; doi:10.1186/1471-2164-11-238)
Supplement: Additional file 4 — Figure S4: gene diagrams showing the contents of all 25 XAC-specific regions. [file 1471-2164-11-238-S4.PPT]

## Slide 1
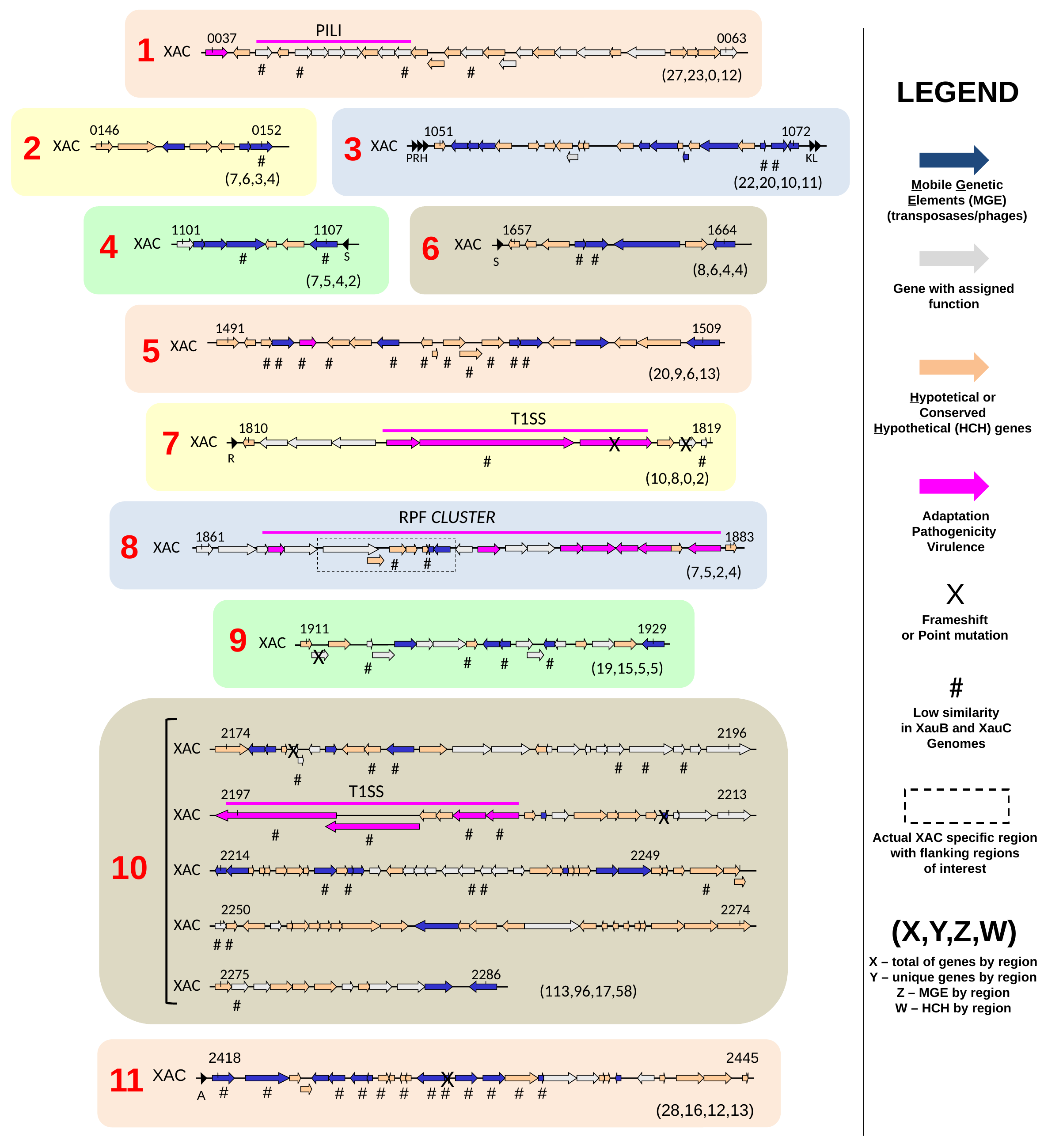

PILI
 0037 0063
1
XAC
#
#
#
#
(27,23,0,12)
LEGEND
 0146 0152
2
XAC
#
(7,6,3,4)
 1051 1072
3
XAC
PRH
KL
#
#
(22,20,10,11)
Mobile Genetic
Elements (MGE)
(transposases/phages)
 1101 1107
4
XAC
#
#
S
(7,5,4,2)
1657 1664
6
XAC
# #
S
(8,6,4,4)
Gene with assigned
function
 1491 1509
5
XAC
# # #
# # #
# # # #
 #
(20,9,6,13)
Hypotetical or
Conserved
Hypothetical (HCH) genes
T1SS
1810 1819
7
X
X
XAC
R
#
#
(10,8,0,2)
RPF CLUSTER
Adaptation
Pathogenicity
Virulence
8
1861 1883
XAC
#
#
(7,5,2,4)
X
Frameshift
or Point mutation
9
1911 1929
XAC
X
#
 #
#
#
(19,15,5,5)
#
Low similarity
in XauB and XauC
Genomes
2174 2196
X
XAC
# # #
 # #
#
T1SS
2197 2213
X
XAC
# #
#
#
2214 2249
10
XAC
# #
 # #
#
2250 2274
XAC
# #
2275 2286
XAC
(113,96,17,58)
#
Actual XAC specific region
with flanking regions
of interest
(X,Y,Z,W)
X – total of genes by region
Y – unique genes by region
Z – MGE by region
W – HCH by region
2418 2445
11
XAC
X
#
#
 # # # # # # # # # #
A
(28,16,12,13)

## Slide 2
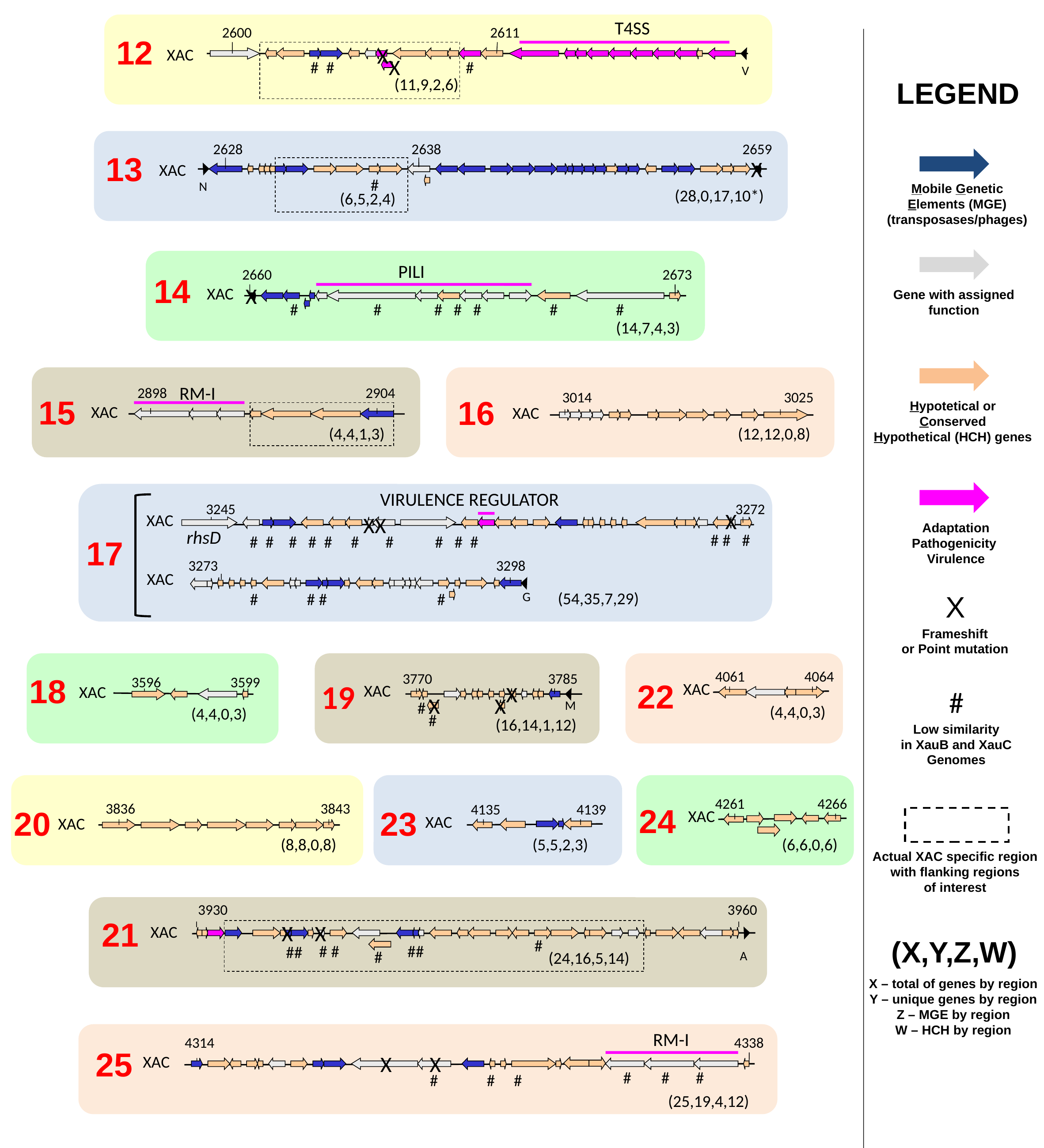

T4SS
2600 2611
12
X
XAC
X
# #
#
V
(11,9,2,6)
LEGEND
2628 2638 2659
13
X
XAC
#
N
(28,0,17,10*)
(6,5,2,4)
Mobile Genetic
Elements (MGE)
(transposases/phages)
PILI
2660 2673
14
X
XAC
#
# # # #
#
#
(14,7,4,3)
Gene with assigned
function
RM-I
2898 2904
15
XAC
(4,4,1,3)
3014 3025
16
XAC
(12,12,0,8)
Hypotetical or
Conserved
Hypothetical (HCH) genes
VIRULENCE REGULATOR
3245 3272
X
XAC
XX
Adaptation
Pathogenicity
Virulence
rhsD
# # #
# # # # # # # # # #
17
3273 3298
XAC
(54,35,7,29)
X
G
# # #
#
Frameshift
or Point mutation
4061 4064
22
XAC
(4,4,0,3)
3770 3785
19
XAC
X
X
X
#
M
#
(16,14,1,12)
18
3596 3599
XAC
#
(4,4,0,3)
Low similarity
in XauB and XauC
Genomes
4261 4266
24
XAC
(6,6,0,6)
3836 3843
20
XAC
(8,8,0,8)
4135 4139
23
XAC
(5,5,2,3)
Actual XAC specific region
with flanking regions
of interest
3930 3960
21
X
X
XAC
#
# #
##
##
#
(24,16,5,14)
A
(X,Y,Z,W)
X – total of genes by region
Y – unique genes by region
Z – MGE by region
W – HCH by region
RM-I
4314 4338
25
X
X
XAC
# # #
# # #
(25,19,4,12)
