# Supplementary figures and images for: Novel insights into the genomic basis of citrus canker based on the genome sequences of two strains of Xanthomonas fuscans subsp. aurantifolii
Source: BMC Genomics. 2010 Apr 13;11:238. doi: 10.1186/1471-2164-11-238 (PMC2883993; doi:10.1186/1471-2164-11-238)

## Slide 1
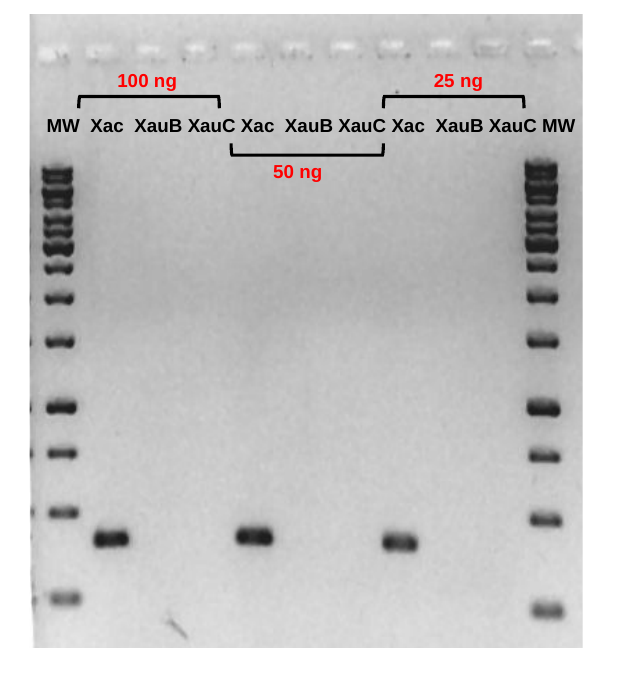

100 ng
25 ng
MW Xac XauB XauC Xac XauB XauC Xac XauB XauC MW
50 ng

Supplement: Additional file 5 — Figure S5: PCR results for gene xacPNP. [file 1471-2164-11-238-S5.PPT]

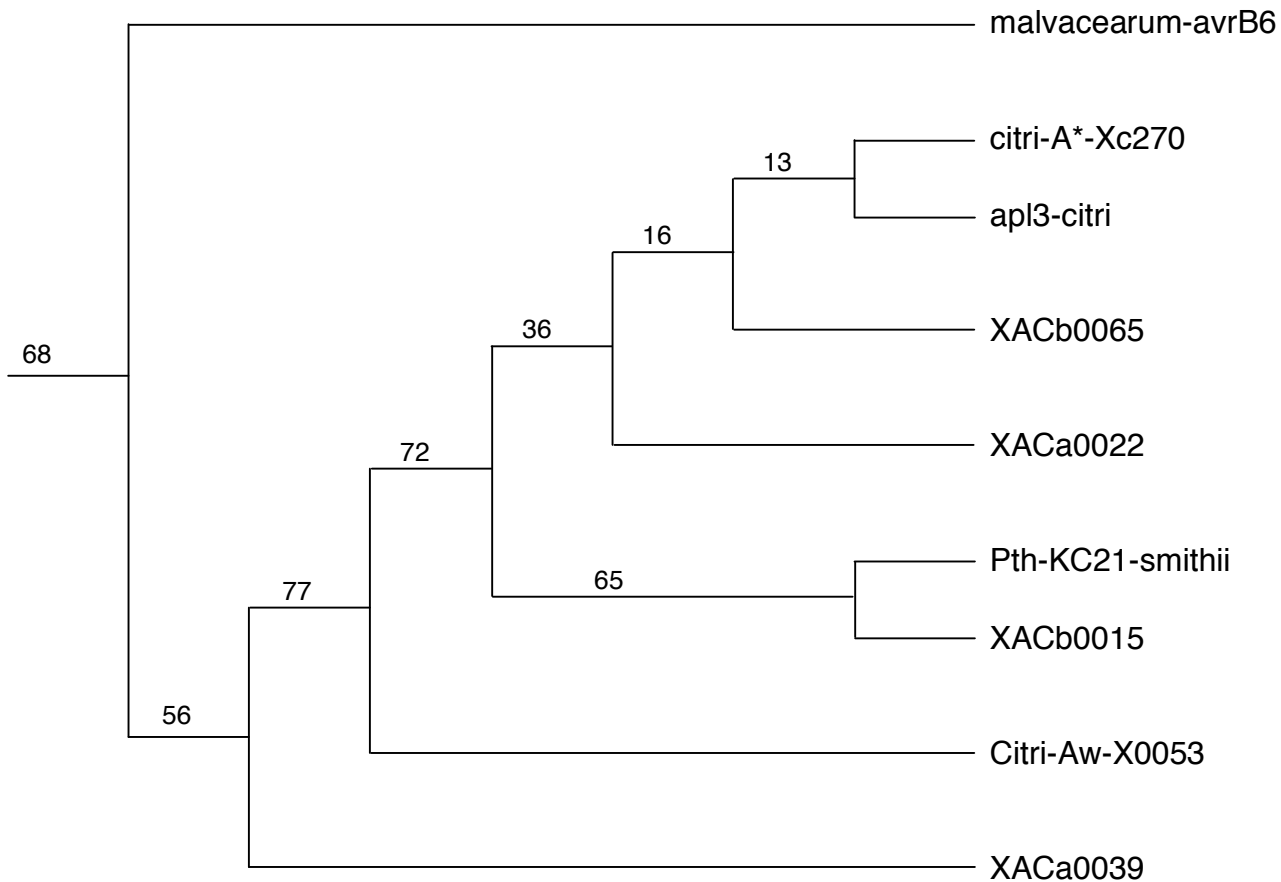

Topology and bootstrap values only; branch lengths do not reflect phylogenetic distance.

Supplement: Additional file 7 — Figure S7: topology of clade in Fig.3with bootstrap values. [file 1471-2164-11-238-S7.PDF]
