# Supplementary material for: Novel insights into the genomic basis of citrus canker based on the genome sequences of two strains of Xanthomonas fuscans subsp. aurantifolii
Source: BMC Genomics. 2010 Apr 13;11:238. doi: 10.1186/1471-2164-11-238 (PMC2883993; doi:10.1186/1471-2164-11-238)
Supplement: Additional file 6 — Table S6: names, organisms, locus tags, and accession numbers for the nucleotide sequences used to build the pth gene phylogeny (Fig.3). [file 1471-2164-11-238-S6.DOC]

**Additional file 6** – Table S6: gene sequences (nucleotides) used to build the *pth* gene phylogeny.

| **organism** | **Gene** | **NCBI accession number** | **NCBI GeneID** | **label in tree** |
| --- | --- | --- | --- | --- |
| XAC | pthA1_XACa0022 |  | 1158467 | XACa0022 |
| XAC | pthA2_XACa0039 |  | 1158484 | XACa0039 |
| XAC | pthA3_XACb0015 |  | 1158502 | XACb0015 |
| XAC | pthA4_XACb0065 |  | 1158552 | XACb0065 |
| XauB | XAUB_40130 |  |  | XAUB_40130 |
| XauB | XAUB_28490 |  |  | XAUB_28490 |
| XauC | XAUC_22430 |  |  | XAUC_22430 |
| XauC | XAUC_24060 + XAUC_09900 |  |  | XAUC_24060_09900 |
| *X. campestris* | pthN | AF016221 |  | malvacearum-pthN |
| *X. campestris pv. malvacearum* | avrB6 | L06634 |  | malvacearum-avrB6 |
| *X. citri pv. citri* strain X0053 | PthAW | EF473085 |  | citri-Aw_X0053 |
| *X. gardneri* | avrHah1 | EF436255 |  | gardneri |
| *X. oryzae pv. oryzae* KACC10331 | pthA1 | NC_006834.1 | 3262051 | pthA1-Xoo |
| *X. oryzae pv. oryzae* KACC10331 | pthA4 | NC_006834.1 | 3265873 | pthA4-Xoo |
| *X. oryzae pv. oryzae* KACC10331 | pthA2 | NC_006834.1 | 3264948 | pthA2_Xoo |
| *X. oryzae pv. oryzae* KACC10331 | avrB6 | NC_006834.1 | 3263649 | avrB6_Xoo |
| *X. oryzae pv. oryzae* KACC10331 | pthA3 | NC_006834.1 | 3263519 | pthA3_Xoo |
| *X. citri* | apl3 | AB021365 |  | apl3-citri |
| *X. campestris pv. armoraciae* | Hax2 | AY993937 |  | Hax2_ armoraciae |
| *X. citri* pv. *citri* Xc270 | pthA* | EF473086 |  | citri-A*-Xc270 |
| *X. axonopodis manihotis* pBsF2 | pthB | AF012325 |  | manihotis-pBsF2 |
| *X. smithii subsp. citri* | pthA KC21 | AB206388 |  | pthA-KC21_smithii |
| *Ralstonia solanacearum* | AVRBS3-LIKE PROTEIN | CAD15517.1 | 17428832 |  |
